# A Portrait of the OPE as a Biological Agent

**DOI:** 10.3390/molecules26113088

**Published:** 2021-05-21

**Authors:** Chiara Maria Antonietta Gangemi, Anna Barattucci, Paola Maria Bonaccorsi

**Affiliations:** Dipartimento di Scienze Chimiche, Biologiche, Farmaceutiche ed Ambientali (ChiBioFarAm), Università degli Studi di Messina, 98168 Messina, Italy; chigangemi@unime.it (C.M.A.G.); pbonaccorsi@unime.it (P.M.B.)

**Keywords:** oligophenylene ethynylenes, luminescent probe, antimicrobial, biocides

## Abstract

Oligophenylene ethynylenes, known as OPEs, are a sequence of aromatic rings linked by triple bonds, the properties of which can be modulated by varying the length of the rigid main chain or/and the nature and position of the substituents on the aromatic units. They are luminescent molecules with high quantum yields and can be designed to enter a cell and act as antimicrobial and antiviral compounds, as biocompatible fluorescent probes directed towards target organelles in living cells, as labelling agents, as selective sensors for the detection of fibrillar and prefibrillar amyloid in the proteic field and in a fluorescence turn-on system for the detection of saccharides, as photosensitizers in photodynamic therapy (due to their capacity to highly induce toxicity after light activation), and as drug delivery systems. The antibacterial properties of OPEs have been the most studied against very popular and resistant pathogens, and in this paper the achievements of these studies are reviewed, together with almost all the other roles held by such oligomers. In the recent decade, their antifungal and antiviral effects have attracted the attention of researchers who believe OPEs to be possible biocides of the future. The review describes, for instance, the preliminary results obtained with OPEs against severe acute respiratory syndrome coronavirus 2, the virus responsible for the COVID-19 pandemic.

## 1. Introduction

The idea of using aryl and ethynyl groups in an alternating fashion to build polymers such as polyphenylene ethynylenes (PPEs) and their corresponding oligomers (OPEs) was introduced in the 1980s, and this led to the discovery of a class of compounds with fascinating properties as materials in different areas, such as sensors, light-emitting devices, and polarizers for LC displays. The polymeric PPEs consist of mixtures of molecules with a broad size range and with no perfect control of sequences; moreover, PPEs tend to form aggregates easily through intra- and/or interchain stacking, in turn reducing their solubility and thus their application in an aqueous environment [1].

For the mentioned reasons, in the last decades the attention of the scientific community has been focused mainly on oligophenylene ethynylenes (OPEs) and their peculiar characteristics and applications in the biological field. The rigid control of the chain lengths in OPEs implies well-defined chemical and physical properties (e.g., solubility), thus allowing for precise interpretations of their actions in biology and definite applications in medical fields.

OPEs can be designed with a controlled monomer sequence, which represents a key feature for their application in biological processes, where the activity of a cell is dramatically influenced by very small environmental and structural changes. Due to their stable π-conjugated rigid skeleton, OPEs are luminescent dyes, with high quantum yields. The presence of aromatic rings linked by triple bonds guarantees a high electron conjugation and, consequently, a prominent absorption of λ_max_ at ~400 nm, which can be tuned by varying the number of aromatic units and/or the substituents. In particular, a larger number of aromatic rings and the presence of donor substituents usually lead to bathochromic shifts, while the presence of acceptor groups seems to be less effective in changing the absorption properties of OPEs. Their emission properties can be modulated by modification of the structure. In the interaction with an analyte or with target molecules, even little changes in the structure (length or monomer compositions) can deeply influence the spectral outputs, allowing us to use them as sensors or as probes. They can be easily differently functionalized by inserting various side chains and/or end groups and numbers of repeating units; this offers us useful spectral information due to several interaction mechanisms with the target (e.g., columbic and/or hydrophobic interactions, solvent quenching effects, and variation of rotational freedom in the backbone). The linear repetitive chain of phenylene ethynylenes is usually decorated at the two extremities (these OPEs are usually called end-only OPEs) or at the core of symmetric ones (these OPEs are sometimes called S-OPEs). Some of them possess a core different from a benzene ring, such as a thienyl moiety (these compounds are sometimes called oligoarylene ethynylenes, OAEs). Unsymmetrically substituted OPEs bear two different substituents attached at the two ends of the main oligomeric chain.

## 2. Synthesis of OPEs

When readers approach the synthesis of OPEs, they need to refer to the synthesis of PPE, at least for what it is concerned with as noted in past literature. The first synthetic procedure for the polymerization of the aryl-ethynyl moiety was described by Lakmikantham et al. in 1983 [2] and was performed via a cuprous acetylide. From that time, more convenient methodologies have been applied, and almost all of them are based on the palladium-catalyzed, cross-coupling reaction of Heck–Cassar–Sonogashira (referred to as Sonogashira reaction or coupling from now on). In a review on the synthesis, properties, structure, and applications of PPE—a work that represents a landmark for many researchers who approached the synthesis of PPEs and OPEs—Bunz [1] gave a detailed and critical discussion on the conditions of such reaction, with respect to the Pd catalyst, the aryl halogen, and the alkynyl derivatives, the base, and the solvent.

In general, sequence-defined oligomers, such as OPEs, can be synthesized by iterative processes in which the coupling of protected monomers, followed by deprotection, are steps that are repeated cyclically until the desired oligomer is obtained. Three main approaches to this general process have been investigated in literature: the solution synthesis, the soluble support-based synthesis, and the solid-phase synthesis [3]. Although the last two methodologies appear the most convenient ones, in terms of purification steps and final yields, classical solution chemistry is the most applied and the one we discuss in this section.

**Scheme 1:**
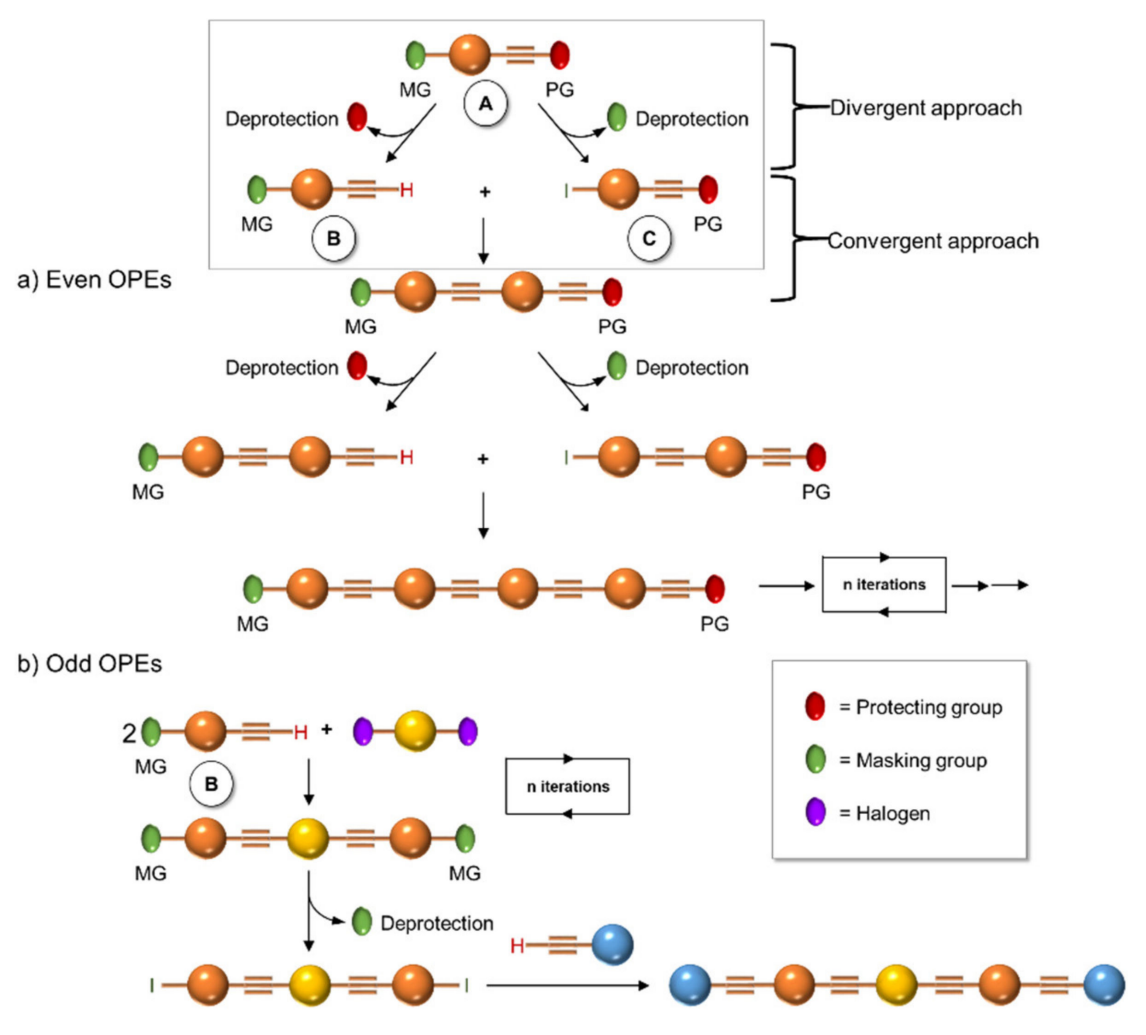
Schematic representation of iterative divergent/convergent synthetic approaches for even (**a**) and odd (**b**) OPEs.

In 1994, Tour et al. [4] presented an iterative divergent/convergent approach to the synthesis of OPEs that has been applied afterwards to the preparation of such molecular wires by several other authors [5,6]. The procedure implies, as a fundamental step, the synthesis of a monomer, such as **A** (Scheme 1), consisting of a protected ethynyl group (PG) linked to an aryl moiety, which in turn possesses in *para* position a masking group of iodide (MG). Each of these protecting groups was removed alternatively in the presence of the other, and the two new monomers **B** and **C** were coupled with each other, doubling the length of the molecule. Successive selective deprotections that were followed by dimerization allowed for the access to oligomers with an even number of phenylene ethynylene moieties (Scheme 1a). The same synthetic approach was used by Moggio et al. [7,8] for the preparation of three families of odd side-chain-substituted OPEs (Scheme 1b), two of which were studied as mesomorphic materials. Compound **1** in Table 1, belonging to the most recent family of R-terminated (dodecyl)benzoateethynylene oligomers, was applied in the staining of bacteria [9].

**Scheme 2:**
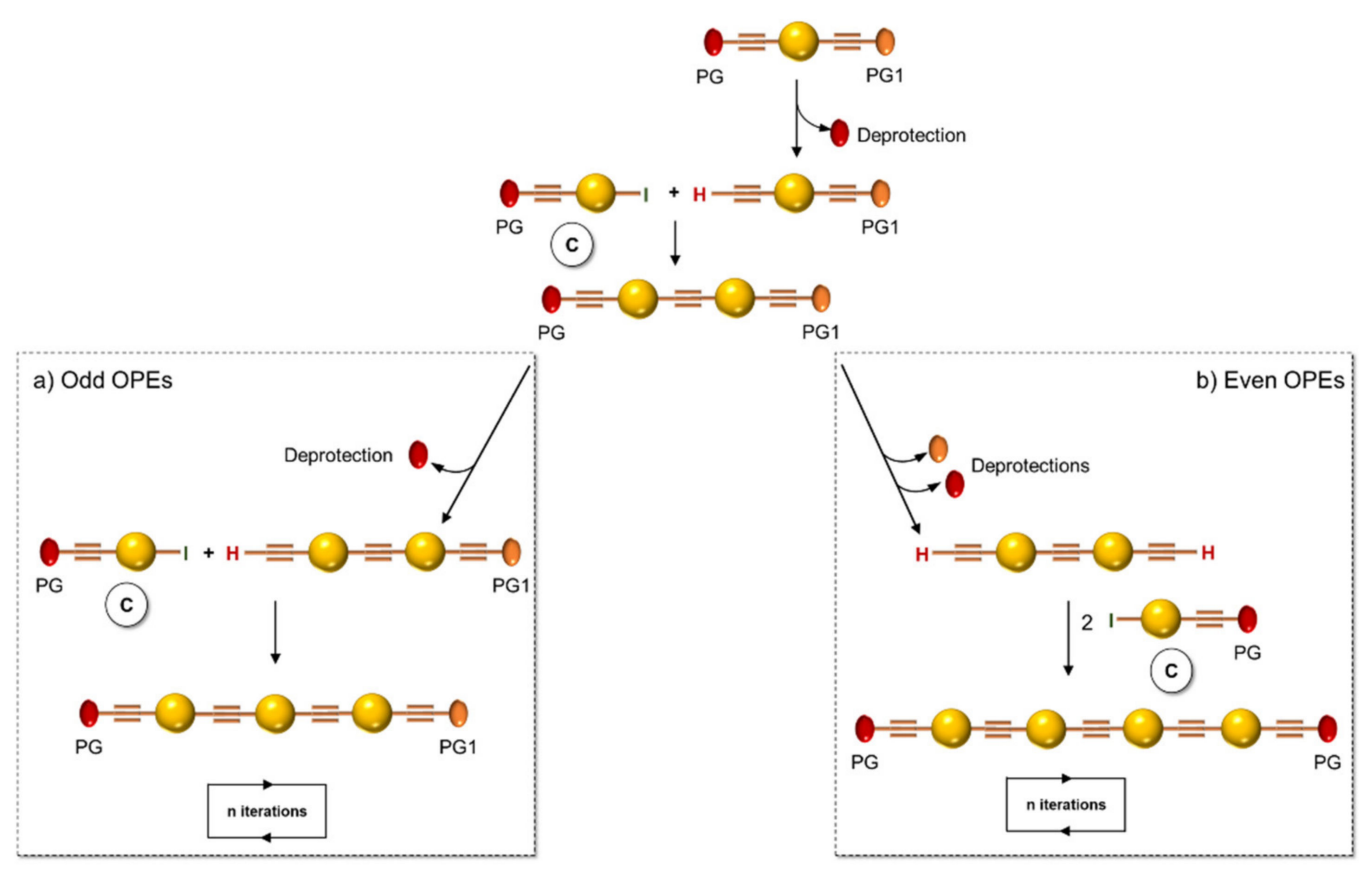
Schematic representation of iterative divergent/convergent synthetic approach by Pertici et al. [5].

Inspired by the iterative divergent/convergent approach, Pertici et al. [5] used as the starting brick of their syntheses a *para*-substituted bis-ethynylbenzene possessing two different protecting groups (PG and PG1) of the triple bond (Scheme 2). In playing on the different degrees of lability of the protecting groups for selective removal and doubling the Sonogashira reactions with the opportunely substituted monomer **C**, the authors afforded symmetrical even and odd OPEs with terminal triple bonds. The obtained oligomers were converted by the copper(I)-catalyzed azide–alkyne cycloaddition (CuAAC) into pseudo-disaccharides. In particular, **2** (Table 1) was used for the inhibition of lectin A from *Pseudomonas aeruginosa* (*P. aeruginosa*) [5,6].

**Scheme 3:**
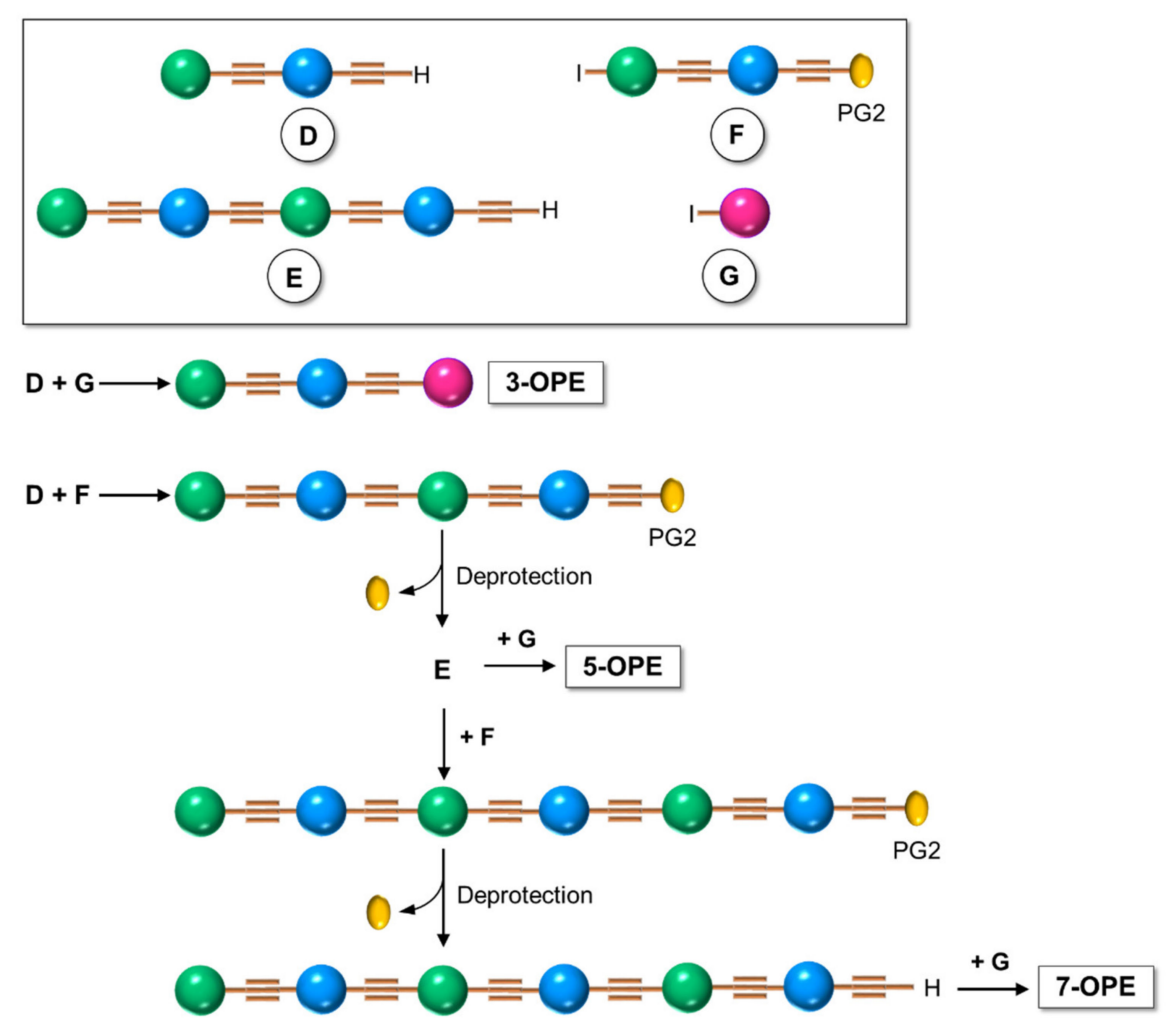
Schematic representation of the step-by-step synthetic approach for unsymmetrical OPEs.

**Scheme 4:**
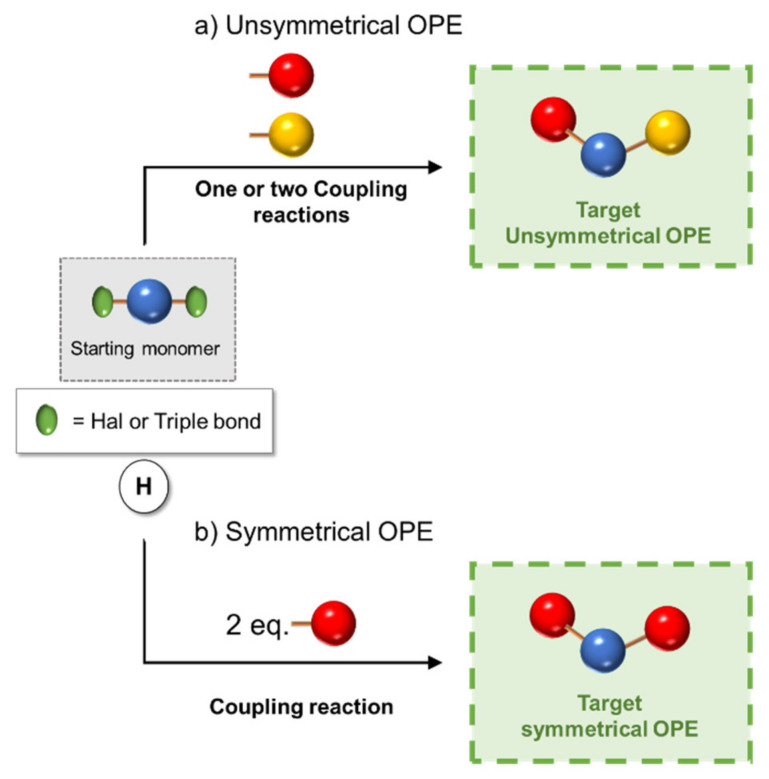
Schematic representation of (**a**) unsymmetrical and (**b**) symmetrical 3-OPEs synthetic approach.

Although the iterative divergent/convergent approach appears effective in the synthesis of OPEs, not all of them can be obtained by this route. The step-by-step solution synthesis of the OPE skeleton, conducted by a series of Sonogashira cross-couplings, in which an opportunely substituted arylalkyne was attached to a conveniently substituted iodobenzene, allowed for the building up of a series of symmetrical and unsymmetrical oligomers with a more original weaving. In particular, unsymmetrical oligomers, where two different substituents are attached at the two ends of the main chain, are the most difficult to be reached: several different building blocks must be prepared and connected to each other (Scheme 3) in a series of frequently low-yielding steps, and the more blocks there are, the longer the OPE’s skeleton is. This is what Whitten et al. [10] described in their last review. Unsymmetrical OPEs containing −(PhC≡C)_n_- chains have been prepared through Sonogashira-coupling of *para*-substituted aryleneethynylenes (such **H** in Scheme 4) with 1-iodo−4-(trimethylsilylethynyl)benzene (such as **C** in Scheme 1), followed by the desilylation and iteration of the coupling, until the desired number of phenylethynylene units was obtained [11].

However, if the oligomer consists of just three repetitive arylethynylene units (3-OPE), the synthesis is concerned with the preparation of a central core, which can be made, for example, of an aryl ring bifunctionalized in *para* position with two halogen atoms (iodine or bromine), and of two para-substituted arylethynylene moieties, different from each other (Scheme 4a). The three building blocks are finally subjected to the Sonogashira cross-coupling reaction, as shown in Scheme 4a [12,13].

Only two building blocks are necessary for the synthesis of symmetrical (identical ends of the oligomer) 3-OPEs: a bifunctionalized aryl core connected with 2 equivalents of a side-armed aryl derivative, both opportunely substituted (Scheme 4b) [14,15].

The symmetrical 3-OPE **3** (Table 1), bearing two 4-aminophenyl-β-D-mannopyranoside end-moieties, was synthesized with the same general approach described in Scheme 4b and used as specific fluorescent marker as well as transducer for the detection of *Escherichia coli* (*E. coli*) [12].

**Scheme 5:**
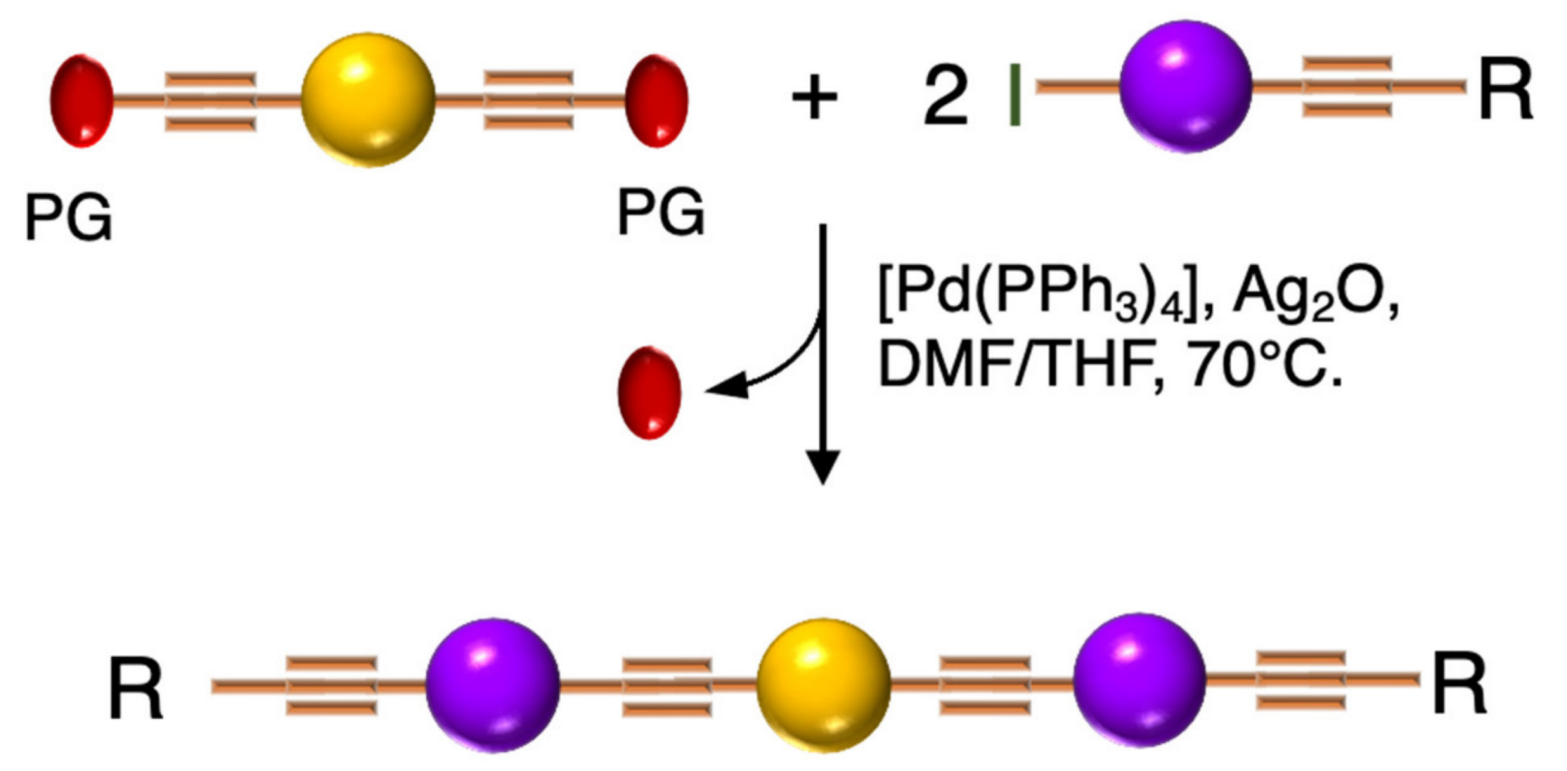
Schematic representation of 3-OPEs synthetic approach via Ag_2_O-based Sonogashira coupling.

Barattucci et al. [15,16,17] reported the synthesis of a series of 3-OPEs (**5**–**10** in Table 1) in which the substituents at the aryl cores are different from those on the aryl moieties of the two side arms, and they also described how such substituents influence the bioaffinity of oligomers under study. The introduction of a NMe_2_ group and its subsequent quaternarization, for instance, led to the preparation of biocompatible probes **7** and **10** [17]. The Sonogashira reaction conditions adopted by these authors consider the use of a trimethylsilyl (TMS)-protected arylethynylene derivative and an aryl iodide, in the presence of a palladium catalyst and silver oxide, as the base, thus avoiding the tedious step of the triple bond deprotection (Scheme 5) [15,16,18].

**Scheme 6:**
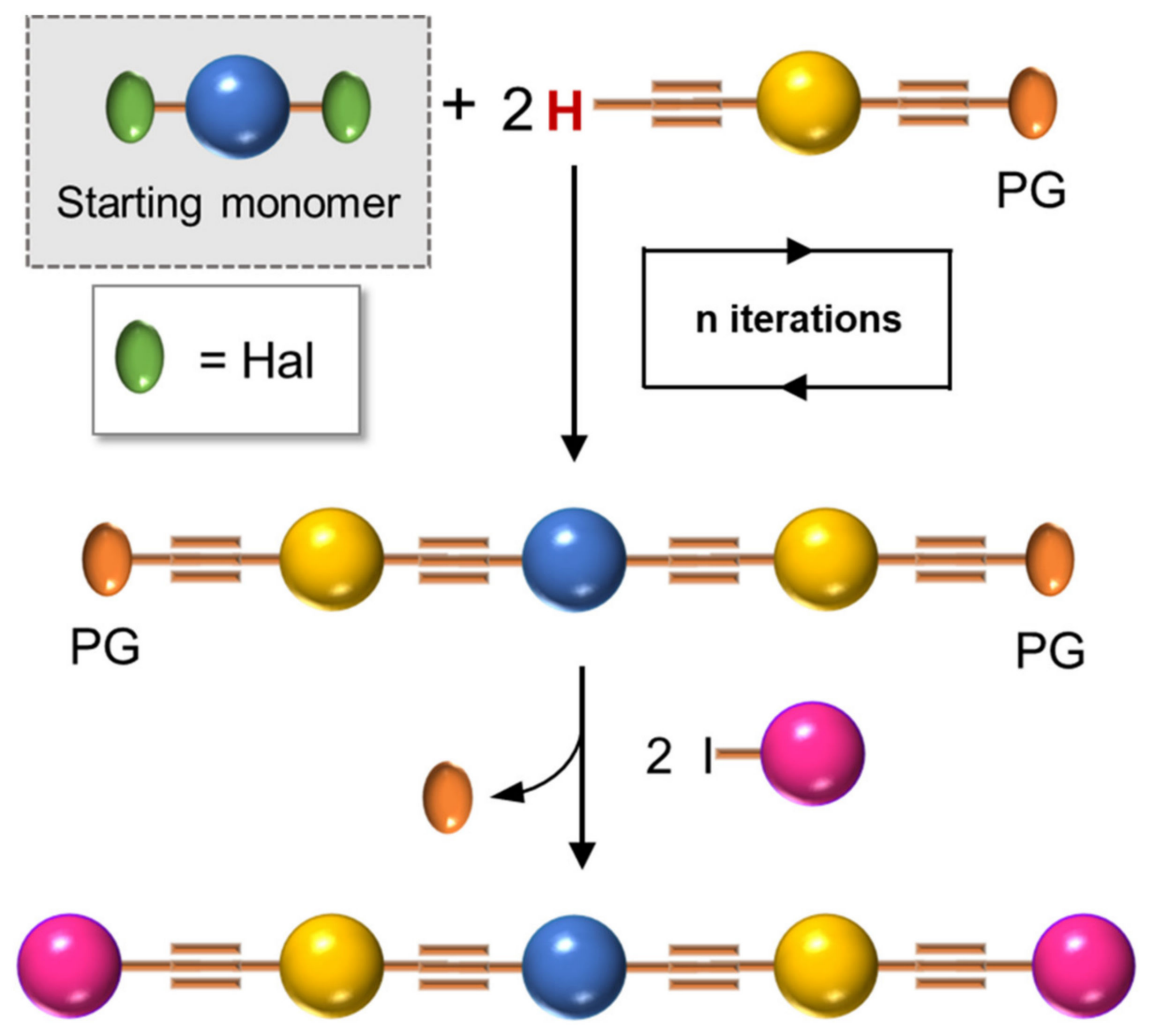
Schematic representation for preparation of symmetrical OPEs longer than three repetitive units.

Finally, most of the preparation of symmetrical oligomers longer than 3-OPEs involves the use of several synthetic steps, as shown in Scheme 6, where the iteration of protection/deprotection steps and Sonogashira reactions represents, once again, the basis of the synthesis [10].

## 3. Optical Properties of OPEs

In fluorescence bioimaging, after excitation, a molecular probe produces luminescence in the visible or in the near-infrared region of the spectrum; the signal is then collected and elaborated through detection equipment that provides an image of the biological tissues. It represents an important visualizing technique and possesses many advantages in comparison with others, such as radiolabeling or magnetic resonance imaging (MRI), because of its high sensitivity, good spatial/temporal resolution, and low damages done to tissues. Furthermore, the fluorescent probe can be labeled with targeting moieties in order to direct its absorption in definite biological compartments. Depending on the physiological environment, the probe can change its photophysical properties, can be activated [19], and thus be used for biosensing. In the last decades, many elegant fluorescent sensing mechanisms have been developed, and many luminescent systems have been used for fluorescence bioimaging in vitro and/or in vivo [20]: inorganic nanomaterials [21], supramolecular assemblies [22], and organic fluorophores [23]. Among them, organic dyes are widely used as imaging and sensing agents (ISAs) in optical microscopy. Due to their interesting fluorescence features, one of the more intuitive uses of OPEs is their application as an ISA in the biological field, after having overcome any problems of water solubility and cytotoxicity. This section comprises two main subjects: OPEs as probes for targeted imaging and theragnostics and OPEs in the field of biosensing.

### 3.1. OPEs as Probes

A series of differently functionalized OPEs were derivatized with a lysine-reactive N-hydroxysuccinimidyl (NHS) group in order for them to be able to covalently attach to proteins, such as HSA (human serum albumin) acting as highly fluorescent labels [11]. In the study, the authors synthetized and characterized a series OPEs (**11**, Table 1) bearing different functional groups. Among them, **11b** was subjected to cytotoxicity studies on lung cancer cells, and it showed very good IC_50_ values, thus confirming the good biocompatibility of this class of molecules. HSA, the model protein, was labeled with compounds **11a–f**; the OPE-HSA conjugates were investigated by MALDI-TOF mass spectrometry and UV–Vis absorption spectroscopy, and they showed a degree of labeling varying from 10 to 3, with good accordance of results between the two techniques. Interestingly, OPE luminescence, which is very low in aqueous media (DMF/H_2_O and DFM/PBS), dramatically increased after protein conjugation; the labeling was also studied using sodium dodecyl sulfate polyacrylamide gel electrophoresis (SDS-PAGE). When irradiated under UV light, the OPE-HSA conjugates exhibit a green fluorescence, which is correlated to their emission quantum yields. Furthermore, through PAGE studies, no changes in the mobility of HSA were found after functionalization with fluorescent probes. These stimulating results paved the way to use OPEs as label agents also for other biomolecules.

**Figure 1:**
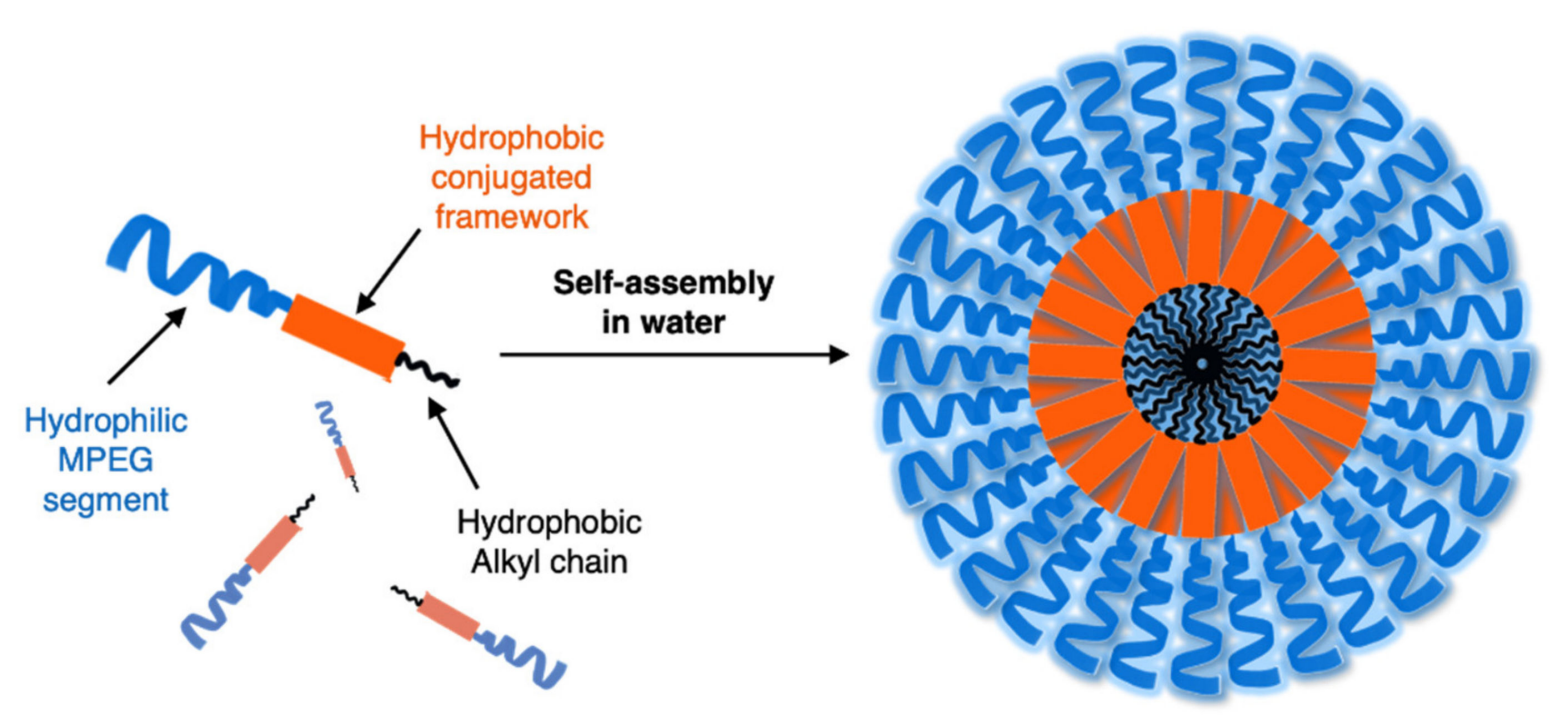
Schematic representation of amphiphilic OPE and its self-assembly into nanoparticles in water.

In 2014 Huang et al. [24] reported the amphiphilic OPE **12** in Table 1, bearing hydrophobic alkyl and methoxy-polyethyleneglycol (MPEG) hydrophilic chains. It can be obtained via Sonogashira coupling (Scheme **4**, unsymmetrical) and is able to self-assemble into nanoparticles (Figure 1) with good water solubility, good stability, and excellent spectroscopic features, useful for cellular imaging through confocal laser scanning microscopy (CLSM) imaging. By varying the lengths of the MPEG pendants (OPE-PEG_350_, OPE-PEG_500_, OPE-PEG_1000_, OPE-PEG_1900_), it was possible to modulate the solubility, morphology, and size of the nanoparticles. The spectroscopic features of all OPE-PEGylated derivatives in THF consist of a strong absorption peak at 330 nm and an emission peak at 425 nm, due to a single OPE molecule. Meanwhile, in water the absorption and fluorescence spectra of OPE-PEG_1900_ **12** (10^−5^ mol L^−1^) suggested the formation of H- and J-aggregates at lower and higher concentrations, respectively, as confirmed by fluorescence lifetime studies. In order to evaluate their applicability for in vivo imaging, the stability of the nanoparticles was tested in calf serum (CS), and no precipitation was observed after centrifugation, even three months later. The photostability was also tested, and after 20 min of UV irradiation, the nanoparticles showed a good stability, probably due to the shielding of the aromatic core from oxygen by MPEG. The cytotoxicity, evaluated in human pancreatic cancer cells (PANC–1) by an MTT cell viability assay, revealed their high biocompatibility, mainly for OPE-PEG_1900_ **12**. A CLSM visualization in vitro, performed after 18 h of incubation, showed a blue fluorescence (410–470 nm) located in the cytoplasm, thus indicating OPE-PEG_1900_ **12** as a good and stable bioimaging cell system.

Huang et al. [25] developed fluorescent water-soluble diamino polyethylene glycol (PEG-NH_2_) OPEs **13** (Table 1), corroborating the ability of such OPE derivatives to self-assemble into nanoparticles with different morphologies, by simply adjusting the concentrations in aqueous solution. The spectroscopic studies of the nanoparticles in water suggested typical J-aggregate behavior. The fluorescence micrograph showed vivid blue emitting nanoparticles well dispersed in water solution, and TEM images showed different morphologies, i.e., grain-like structures at lower concentrations and strawberry-like structures at higher concentrations. The biocompatibility of such OPE-based nanoparticles was tested by an MTT assay on PANC–1 cells, and good cell viability was observed (>90%). Furthermore, live cells imaging after the incubation of OPE **13** was performed by CLSM, and a strong fluorescence at 720 nm was recorded in the cellular cytoplasm, thus providing a long-term and spatially resolved imaging with reduced cell damages. Starting from these interesting results, Huang’s group [26] developed fluorescent magnetic nanoparticles (FMNPs), using iron oxide nanocrystals decorated by amphiphilic PEG-functionalized OPEs. Amino-modified PEG pendants were introduced as a hydrophilic portion (MNPs@OPE-PEG-NH_2_ **14a**, Table 1) and a folate as targeting moiety (MNPs@OPE-PEG-FA **14b**, Table 1). The folate was introduced in the OPE because its receptor is highly expressed on the cell surface of many kind of cancer cells. Such FMNPs were characterized by UV–Vis absorption and photoluminescence spectroscopy, confirming the formation of H-type assemblies on the surface of MNPs. The magnetic resonance analysis showed a very efficient contrast agent ability of MNPs@OPE. The biocompatibility of such MNPs (**14a** and **14b**) was evaluated by an MTT assay using 3T3 fibroblasts, which showed a good viability of the cell treated with both types of nanoparticles. To investigate the targeting ability of the nanoparticles, HeLa cancer cells were incubated with both MNPs, leading to a significant negative contrast enhancement for the magnetic resonance imaging of MNPs@OPE-PEG-FA **14b**. The specific internalization of the nanoparticles was higher for **14b** in the HeLa cells and in particular in the cytoplasm, while for MNPs@OPE-PEG-NH_2_ **14a** a weak fluorescence was recorded in the 3T3 fibroblasts, confirming the targeting ability of folate nanoparticles. The dual imaging ability of MNPs@OPE-PEG-FA **14b** was also tested in vivo, using animal tumor bearing models, and their selective internalization was confirmed by a Prussian blue staining test, with a high quantity of iron in the MNPs@OPE-PEG-FA **14b** treated tissues. Unfortunately, the low emission wavelength (460 nm) prevents their use for fluorescence imaging in vivo, but nevertheless the system can still be used for the optical imaging of tissues in biopsies and thus for postoperative analysis.

Barattucci et al. [15] developed new OPE-glucoside conjugates **5–6** (in Table 1) for cellular uptake and visualization. A series of 3-OPEs that possess acetyl-protected or unprotected β-D-glucopyranose terminations, with differently substituted aromatic cores, were obtained through the general procedure illustrated in Scheme 4. Very significant results were obtained with compound **6a**, where the introduction of a NMe_2_ group in the central aromatic core produced an interesting modulation of the emission profile and improved the internalization performed using human larynx epidermoid carcinoma tissue cells (Hep-2). Moreover, compound **6a** was found localized mainly in the cytoplasm, which allowed for visualization through fluorescence microscopy already at 1 µM of OPE. Furthermore, the protected compound **6c** was slightly internalized in Hep-2 cells. Additionally, the cell viability, tested with trypan blue assay, showed a good biocompatibility of these OPEs, confirming the possibility to use them as modifiable platforms for bioimaging. The presence of one or two positive charges in the dialkylamino-substituted OPEs **7** and **10a** (Table 1) improves water solubility and, in cooperation with “H-bonding” sugar groups, makes them able to interact with DNA [17]. The noncovalent interactions with DNA were characterized by hypochromicity and accompanied by a red and a blue shift for dicationic **10a** and monocationic OPEs **7**, respectively. Circular dichroism studies and the different spectroscopic behaviors have suggested a mixed binding mode of OPEs **7** and **10a** with the biopolymer, ascribable to intercalation (suggested by absorption titrations and melting temperature measurements) and an external interaction (suggested by CD and viscosity results). Thus, it was reported by the authors that the positively charged OPEs **7** and **10a** were first electrostatically attracted by negative DNA backbone and then inserted the aromatic core between DNA bases. At the same time the glucose groups stabilized the interaction through hydrogen bonds. The biocompatibility of OPEs was tested on healthy Vero cells (African green monkey kidney cells) and Hep-2 cancer cells; no toxicity for OPEs was found in the healthy cells, and a reduction of cellular proliferation was seen in the Hep-2 cell for the dicationic OPE **10a**. This result was attributed to a different cellular uptake process with respect to monocationic OPE **7**, joined to the more pronounced mitosis in cancer cells, with the exposure of genetic materials, which supports the higher sensitivity of OPE **10a** to Hep-2 with respect to healthy cells.

**Figure 2:**
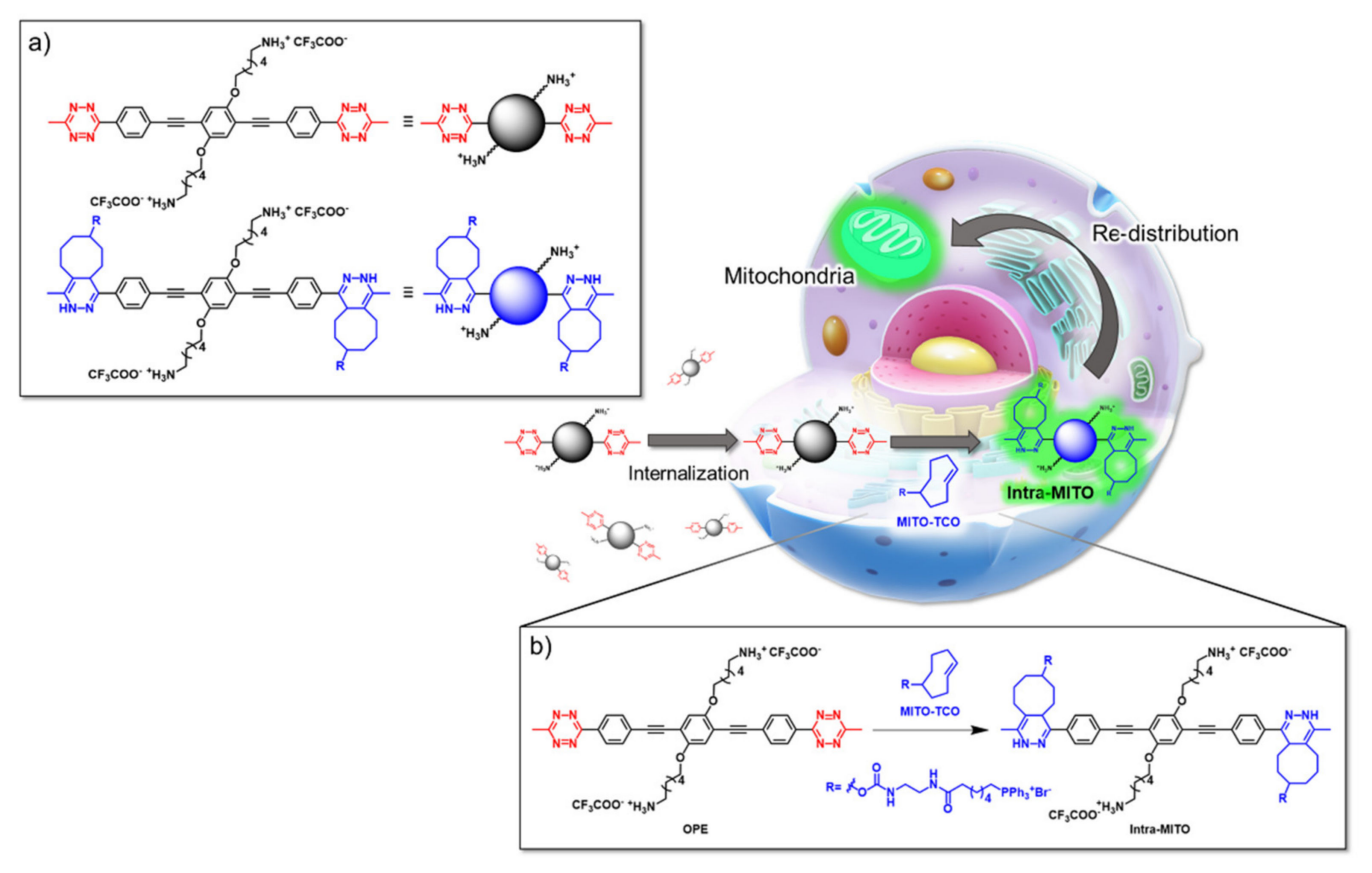
Schematic illustration of the mitochondria-targeting process of tetrazine-conjugated OPE **15** in living cells: (**a**) the simplified representation of OPEs **15** and **16**; (**b**) the intracellular synthesis of fluorescent intra-MITO.

An innovative imaging system, one based on orthogonal reactions that take place in a biological environment, was developed by Wang et al. in 2018 [27]. They used modified OPEs **15** and **16** (Table 1) to target mitochondria through “bioorthogonal reactions”. Mitochondria are very important organelles that are present in the eukaryotic cells, and they are involved in energy production and in many other biological processes. In OPE **15**, the presence of tetrazine induced a turn-off of the luminescence through bond-energy transfer (TBET). When the tetrazine-substituted OPE **15** reacts, inside the cell, in a “bioorthogonal reaction” with an opportune dienophile synthesized by the authors (MITO-TCO) and containing a tri-phenyl phosphine moiety (TPP), the intra-MITO derivative **16** is obtained and the fluorescence turns on, allowing for the visualization of the cell compartments (Figure 2b). By CLMS measurements, it was possible to confirm a good overlap between intra-MITO **16** (blue fluorescence) and the dye MITO-tracker (red fluorescence), confirming the good targeting ability of the TPP group, after bioorthogonal intracellular reaction. The realized system was able to be directed towards mitochondria and visualize them, offering a guide also for other kinds of organelles in living cells.

**Figure 3:**
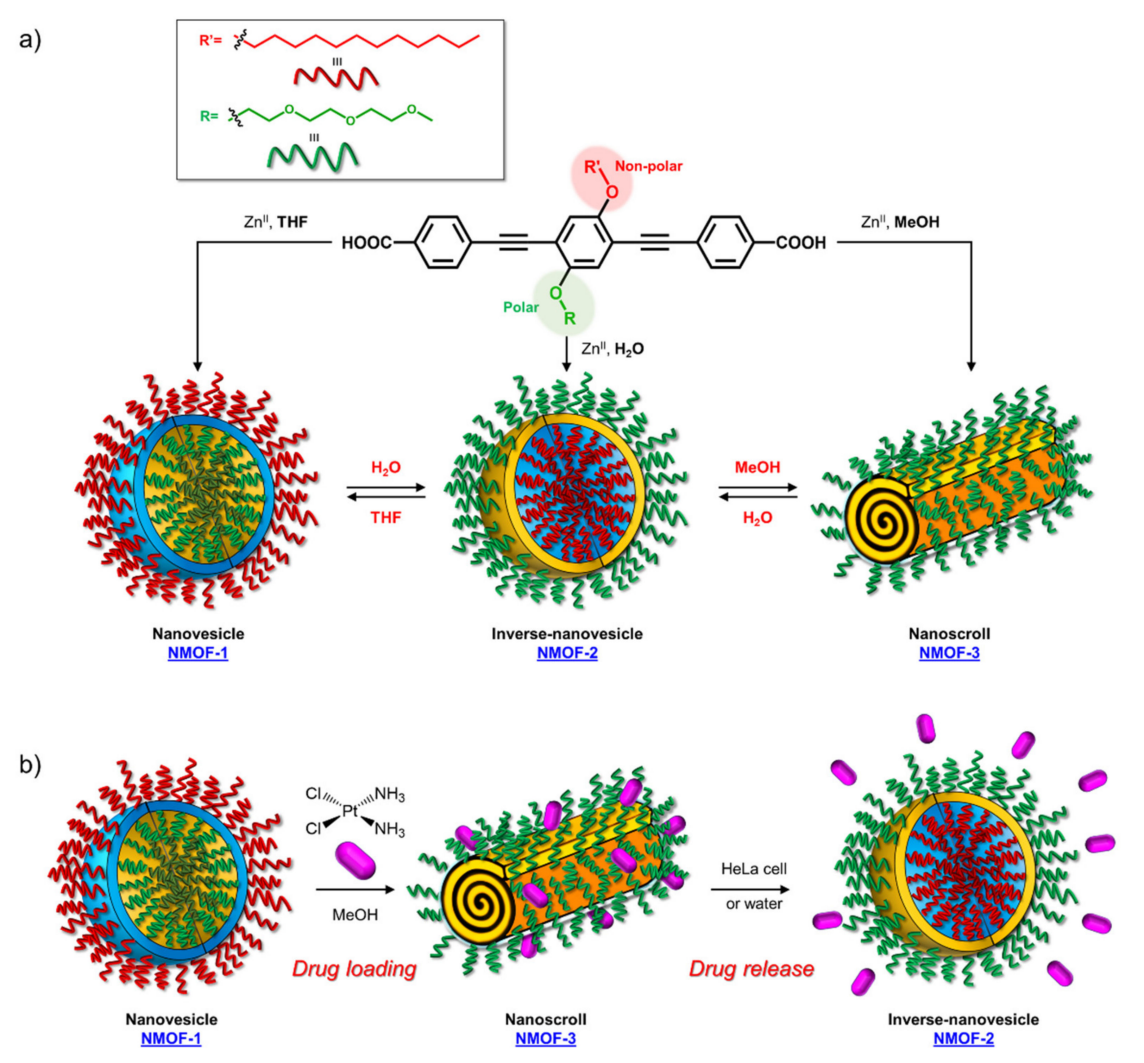
Schematic representation of (**a**) synthesis and morphology of NMOFs and (**b**) drug loading and release of cisplatin.

The possibility of using OPE derivatives for dual imaging and drug-carrying systems represents a goal for many scientists. In 2019, Maji et al. [28] developed a metal organic hybrid system for imaging and drug delivery that is based on functionalized OPEs possessing solvent-adaptive behavior. These oligomers form a nanoscale metal organic framework (NMOF) with different morphologies, depending on the solvent (Figure 3a). In particular, carboxylic functionalized OPE **17** (Table **1**), used as chelating for Zn(II), was decorated with alkyl and glycol chains and was able to form three nontoxic and reversibly shaped NMOFs: nanovesicle (NMOF-1), inverse nanovesicle (NMOF-2), and nanoscroll (NMOF-3). Since the NMOF was assembled through hydrogen bonds and the π-π interaction between PEG and alkyl chains, respectively, the authors tried to change the shape of the nanosystem by varying the solvent polarity. Interestingly, the morphology of the system can be modulated by passing through THF→H_2_O→methanol, in turn obtaining NMOF-1→NMOF-2→NMOF-3, in a reversible fashioned way. In addition, all systems showed an OPE typical cyan emission (43% of quantum yield) and thus were used for living cell bioimaging on HeLa (human cervical cancer) cells. After treating HeLa cells with NMOF-1, a cyan emission in structured illumination microscopy (SR-SIM) was recorded, confirming its efficient uptake. Considering the good ability of internalization of NMOF-1, the nanovesicles were loaded with cisplatin (cisplatin@NMOF-1 loading amount of 14.4 wt%). The loading was monitored by different techniques, which suggests a change in the morphology of a cisplatin-loaded system when passing from NMOF-1 to NMOF-3 shape, with cisplatin homogeneously encapsulated in the nanoscroll (Figure 3b). In vitro release studies showed a release of drugs from 67% up to 82% with a good stability of NMOF. Furthermore, the in vitro studies on HeLa cells incubated with cisplatin@NMOF-1 and with NMOF-1 as control showed a high toxicity (IC_50_ = 0.5 μM) for cisplatin@NMOF-1 and no toxic behavior for the control, confirming the good ability of NMOF as a nanocarrier and delivery system. Similar studies were conducted with doxorubicin (DOX), a commercial anticancer drug: in this case, although it had a lower loading capability (4.1 wt%), the release was of 99%. According to these studies, the innovative solvent-adapting nanovesicle system that was developed can represent a very good and versatile drug delivery and bioimaging system.

Another example of fluorescent-imaging molecules that are exploitable for medical application, such as photodynamic therapy (PDT), was reported by Barattucci et al. [16]. Photodynamic therapy is a very useful treatment in which the tumor tissues can be eradicated through the use of a photosensitizer which, after absorption of light, produces, in the presence of oxygen, highly reactive oxygen-based species (ROS) that damage the tumor cell, thereby inducing its death, by different modalities. The ideal photosensitizer should possess a low or absent dark toxicity, a high toxicity after light activation by a selective excitation wavelength, and chemical and physical stability but also a good facility in the distribution and elimination pathway. Encouraged by the good internalization in cells of the end-glucose amino OPEs **6** and **9** (Table 1), Barattucci et al. [15] tested the capability of these systems to work as photosensitizers and thus, based on the previous results, they synthetized 3-OPEs, which end with glucose units and with different functionalization on the aromatic rings for improving biocompatibility and solubility in aqueous media. After spectroscopic characterization, all compounds were tested for singlet oxygen production, through uric acid (UA) as the detector and methylene blue as the reference photosensitizer, thus recording the reduction of absorption of UA in the presence of the different OPEs. No singlet-oxygen production was recorded for the control OPE **5c**. The internalization of **6a** and **9a,** was conducted on HaCaT (immortalized human keratinocytes), HeLa, and Hep-2 cell lines. After the incubation of the two OPEs showed a good internalization, mainly in the Golgi apparatus and in the endoplasmic reticulum, an intense fluorescence allowed for imaging after the uptake, without any different behavior among the cell lines. In addition, none of the OPEs showed dark cytotoxicity at a concentration of 3 µM, while a reduced cell viability (from 60% to 85 %) was found for higher concentrations, which are comparable to other commercial and approved drugs. The cytotoxicity, after UVA irradiation, was evaluated, and the findings highlighted its dependence from concentration and UVA dose, reaching the LD_50_ dose with [OPE]= 3µM. Unfortunately, no selectivity among the tumor cell lines (HeLa and Hep-2) and nontumor line (HaCaT) was found. Studies on the cell morphology after PDT showed that only amino-OPEs cause a damage induced by ROS production in the microtubule network of the cancer cell, which in turn induces an alteration of the metaphase cell cycle. Therefore, these studies represent the first step to develop an OPE-based platform, which is made possible due to the photoluminescence features of OPEs for imaging as well as for PDT and medical applications.

### 3.2. OPEs as Sensors

Another important application of small luminescence molecules is represented by tracking or sensing. Different works have studied the use of OPEs for the detection of nitroaromatics (NACs) [29], as well as their use as a molecular junction for sensing hydrogen gas [30] or metal cations [31], and as a graphene oxide nanocomposite [32] for the recognition of dopamine and cysteine [33].

**Figure 4:**
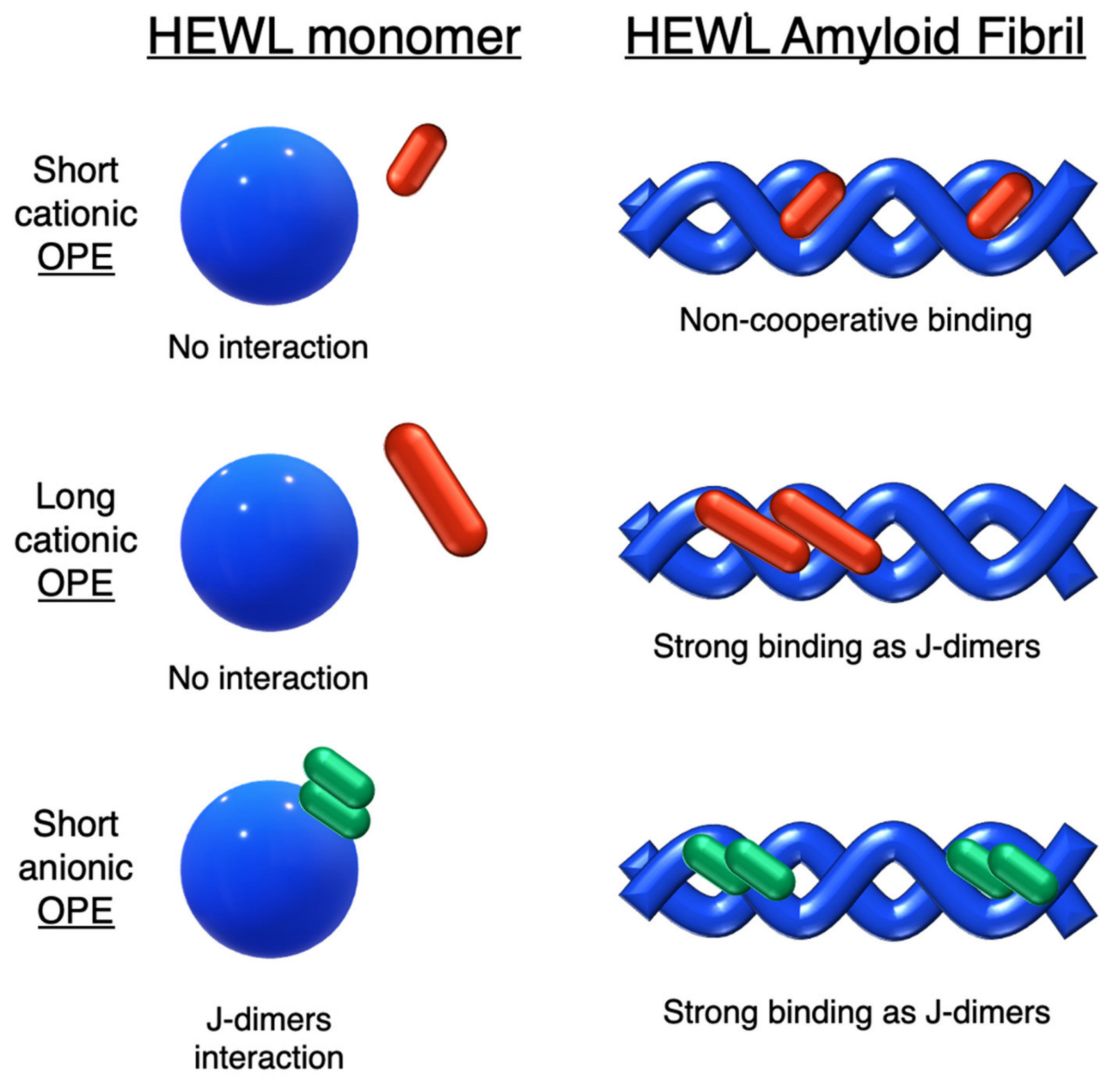
Schematic illustration of binding modes of OPEs **18d**-**f** (in red) and **19c** (in green) with HEWL and HEWL fibrils.

In this field, advances were obtained by Whitten’s research group, who investigated the ability of OPEs to act as sensors for amyloids [34], whose existence is correlated to many neurodegenerative diseases, such as Parkinson’s (PD) and Alzheimer’s (AD) diseases. Common features for amyloid specific sensors are the presence of a linear conjugated and aromatic backbone, which promotes the binding to protein surface through hydrophobic interactions. PPEs were already used as probes for detection of protease [35] and phospholipase C [36] activity studies. In this study, OPEs with ethyl ester termini and of various charges and repeating units were studied as in vitro fluorescent probes for an amyloid model from hen egg white lysozyme (HEWL). Two groups of OPEs were analyzed, i.e., OPEs **18d**-**f** (Table 1) with positive alkyl ammonium pendants and different repeating units and an anionic OPE **19c**, which possesses sulfonated chain groups and a single repeating unit. The selected OPEs were achiral, amphiphilic, and water soluble, and as demonstrated previously [37], they enhanced their fluorescence when bounded to a hydrophobic surface because of a reduced water solvation quenching. All OPEs shown an increase in fluorescence intensity in the presence of amyloid and no fluorescence changes in presence of the monomeric form, except for OPE **19c**. The excitation spectra for all OPEs showed a bathochromic shift in the presence of amyloids and no change in the presence of a monomeric form. Furthermore, the induced circular dichroism (ICD) of an OPE–amyloid complex was analyzed in order to verify whether the intrinsic chirality of the proteins were transferred to the silent OPE chromophores when they are bonded into the complex form. No optical activities were recorded for cationic OPEs **18d**-**f** and **19c** with HEWL monomers; in the presence of HEWL amyloids, silent CD spectra were recorded for **18d**, whereas strong ICD signals were observed for **19c**, **18e**, and **18f**. Thus, according to the experimental results, the authors suggested that OPEs can bind to the proteins as single molecules or as J-dimers. In particular, for **18d** the spectroscopic evidence and the absence of optical activity suggest a noncooperative and saturable binding to the fibrils as single molecules rather than aggregates. Compound **19c** showed a nonspecific weak binding as J-dimers for monomeric proteins, probably due to hydrophobic and electrostatic interactions with the positive lysozyme (as already seen for similar PPEs [38]) and a stronger binding as J-dimers with fibril HEWL causing ICD signal. Finally, the spectroscopic evidence for longer cationic OPE **18e** and **18f** suggested a larger chiral J-aggregation on the amyloids surface (Figure 4). The interesting studies reported by Whitten described how OPEs can be used as selective sensing for amyloids fibril models, thereby exploiting their different spectroscopic behaviors (e.g., enhancement of fluorescence, induced circular dichroism) comparable to Thioflavin T (ThT), the most widely used dye for the detection of amyloids.

**Figure 5:**
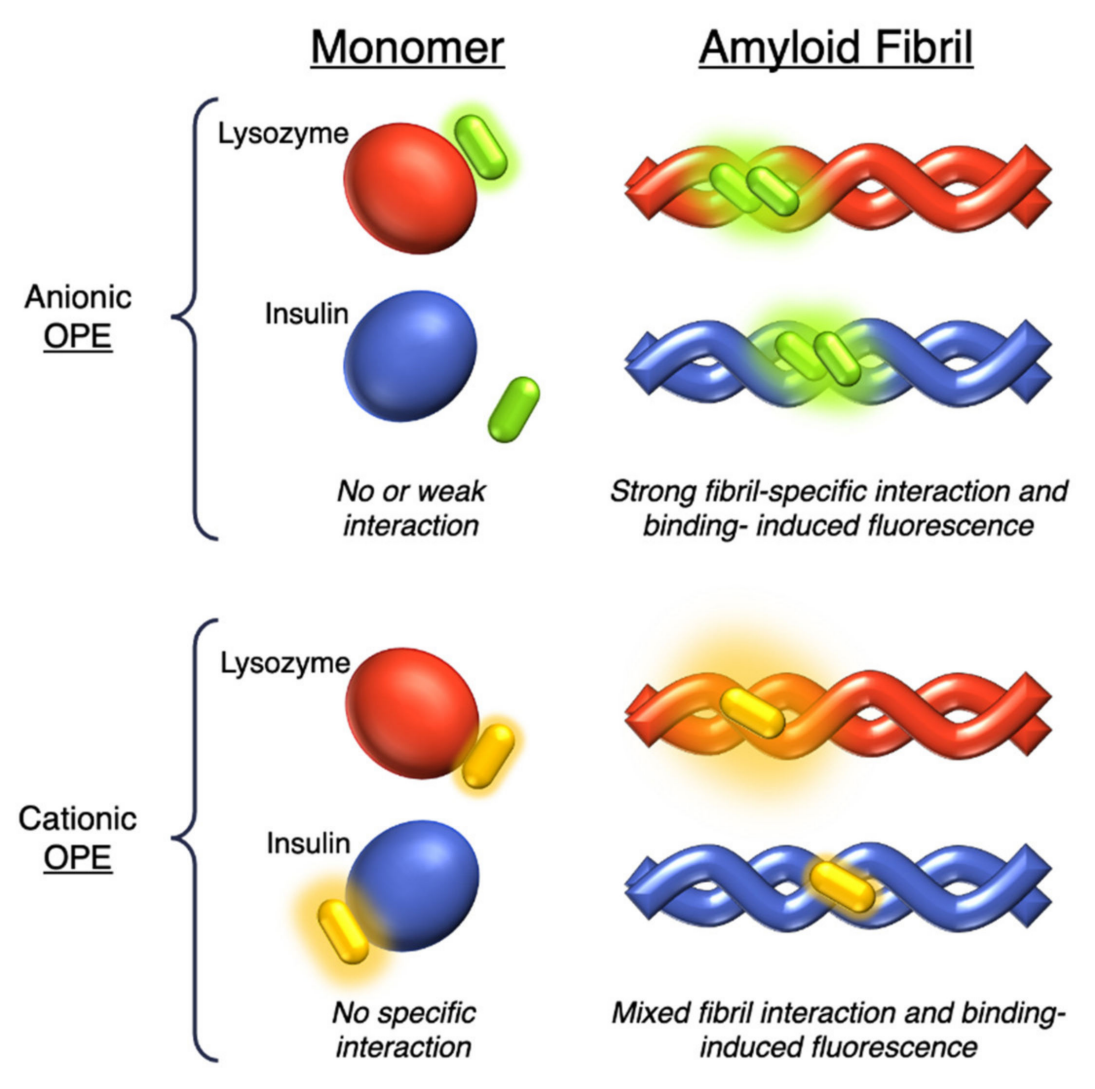
Schematic representation of the sensing modes of anionic **19c** (in green) and cationic **18d**-**f** (in yellow) OPEs towards lysozyme (in red) and insulin (in blue) monomers and fibrils.

The same group in 2017 selected a small library of OPE and PPE derivatives [39] as amyloid trackers towards another amyloid protein model bovine insulin, which, in a fashion similar to the previously studied lysozyme [34], was able to form fibrils in acid and high temperature conditions. Unlike the lysozyme, bovine insulin shows a negatively charged surface at neutral pH. In order to verify the probe ability of these compounds, the excitation and emission fluorescence spectra of each compound were recorded both alone and after incubation with monomeric and fibril forms of HEWL and bovine insulin. Most of the studied compounds showed either a poor binding property toward the two proteins or no changes in fluorescence after interaction and, consequently, the impossibility to use them as sensors. The oligomeric compounds with positive charges at the end of their backbones resulted in them not being usable for sensing. However, some oligomeric positive compounds have a good fibril selectivity, and some others present a monomeric selectivity. In particular, the cationic **18d** and the anionic **19c** (Table 1) can represent selective sensors for fibrils. Almost all active probe compounds showed a common terminal ethyl ester group, which guarantees, as demonstrated elsewhere [37], a fluorescence unquenching after interaction with hydrophobic molecules (such as amphiphiles or proteins), due to the removal of water molecules from a hydration shell. Among the positive ethyl ester terminated dyes, **18e** and **18f** showed an increased fluorescence intensity and thus a better selectivity for lysozyme fibrils over the monomeric form of HEWL. The opposite behavior was detected for insulin. In this case, a more intense fluorescence was recognized for the monomeric forms than fibrils, probably due to a coulombic interaction between the positive dyes **18e**, **18f**, and the negatively charged insulin monomer (Figure 5). Among the anionic OPEs, **19a** and **19b** exploited favorable coulombic interactions with the positive lysozyme surface, but the lack of ethyl ester terminal groups impeded the increasing of fluorescence after the binding event and thus prevents their use as sensors. The best candidate for amyloid sensing was **19c**: it showed an increase of fluorescence intensity in the presence of both lysozyme and insulin fibrils, although a very small fluorescence increase was observed for monomeric forms (Figure 5). This effect may be due to a higher solvation energy of the anionic **19c** with respect to the −NMe_3_^+^ functionalized OPEs. Furthermore, induced CD signals were recorded for **19c** with lysozyme and insulin fibrils, suggesting a chiral J-type dimer binding mode that was more pronounced for insulin. Finally, the probe size plays an important role: the polymeric molecules can largely self-aggregate, thus reducing their solubility and their emission intensity. The smaller OPEs are instead prone to form H-type aggregates with a loss of fluorescence yield. Among the **18d**, **18e**, and **18f**, which differ only in size, **18e** has the best performance; **18d** is too small and does not possess the requested hydrophobic surface to bind to the protein, while **18f** is not able to induce the shielding of water molecules in the ethyl ester terminal group and, because of its longer and rigid backbone, cannot fit well with the protein surface.

**Figure 6:**
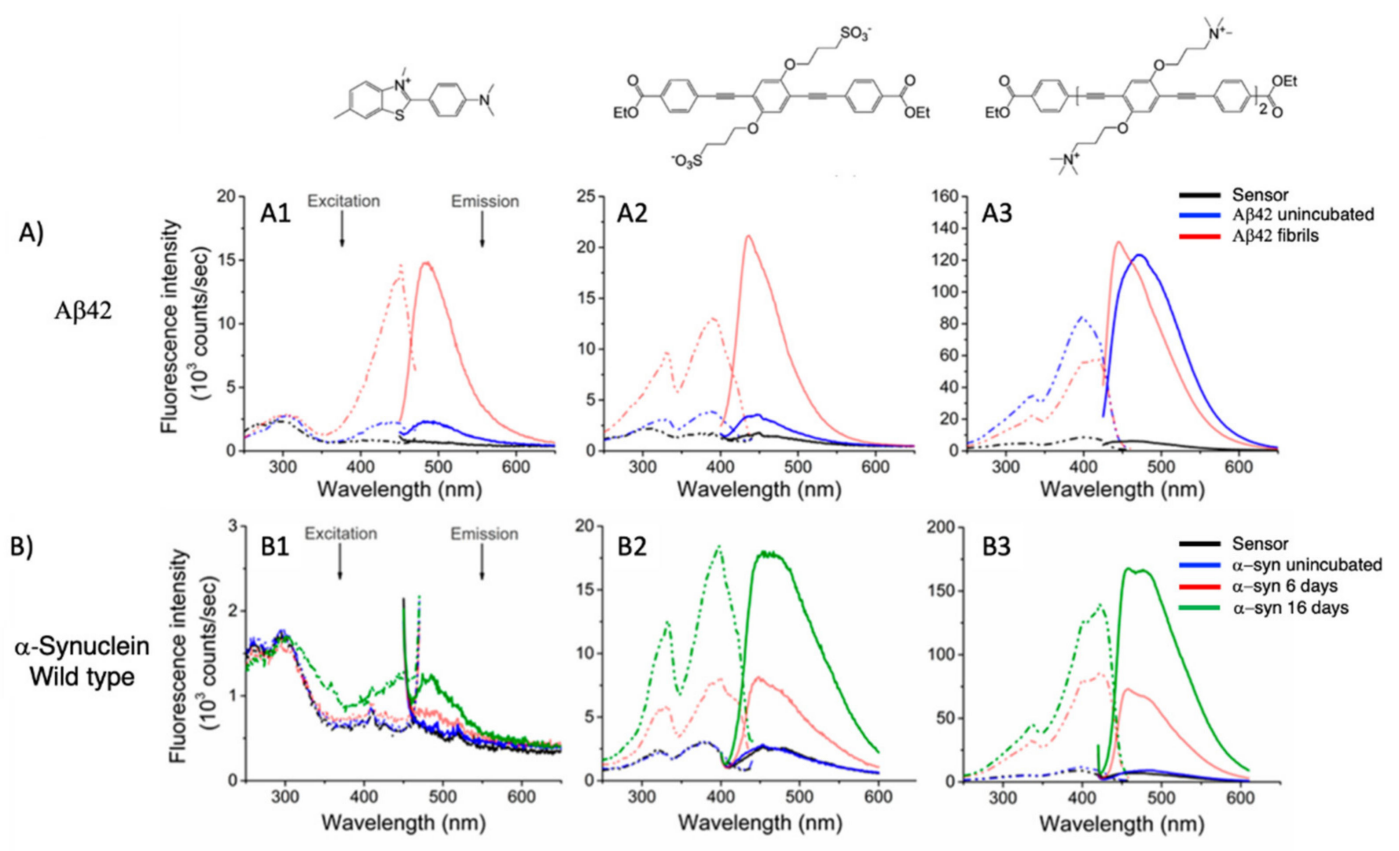
(**A**) Excitation (dashed line) and emission spectra (solid line) of ThT (A1), **19c** (A2), and **18e** (A3) in the presence of Aβ42 (up) alone (black line), in the presence of Aβ42 unincubated (blue line), and in the presence of Aβ42 fibrils (red line). (**B**) Excitation (dashed line) and emission spectra (solid line) of ThT (B1), **19c** (B2), and **18e** (B3) in presence of α-synuclein wild type (bottom) alone (black line), in presence of α-synuclein unincubated (blue line), and in presence of α-synuclein after 6 days of incubation (red line) and after 16 days (green line). Adapted with permission from [40]. Copyright 2019 American Chemical Society.

Very recently, a remarkable improvement in this field was achieved by Chi et al., who used **19c** and **18e** (Table 1) for detecting fibrillar and prefibrillar amyloid proteins [40]. The two OPEs were tested as molecular sensors for fibrillar and prefibrillar aggregates of Aβ40 and Aβ42 peptides associated to AD and for four variants of α-synuclein (wild type and mutants A30P, E35K, and A53T) associated to PD over their monomeric counterparts. To evaluate the binding properties, the excitation and emission spectra of ThT (as a model compound), **19c**, and **18e** in the presence of monomeric and fibrillar Aβ40 were measured. In buffer solution, OPEs showed low fluorescence intensities due to the water quenching effect, and no increase of fluorescence was recorded in the presence of the monomeric form of Aβ40, except for a slight increase recorded for **18e**, probably attributable to the electrostatic interactions. On the other hand, a large fluorescence amplification was produced when OPEs were mixed with Aβ40 fibrils. This behavior was ascribed to the **18e** J-dimer formation by a stacking interaction, to the planarization of an aromatic backbone and the desolvation of ethyl ester terminal groups. Moreover, the ThT/fibril complex showed a fluorescence that is 10 to 30 times lower than the corresponding complexes with OPEs (Figure 6a). According to the spectroscopical results, all the analyzed compounds are selective for fibrils, but OPEs gave the best fluorescence response. Moreover, **18e** seemed to have a higher affinity toward longer fibrils obtained in tris buffer rather than toward the shorter ones in phosphate buffer, while **19c** exhibited a better affinity with shorter fibrils. For these reasons, supplementary binding studies were conducted with Aβ42 and four α-synuclein forms. It was observed that Aβ42 is the more amylogenic form of Aβ proteins, as its deposition starts prior to Aβ40, showing even for unincubated Aβ42 the presence of oligomeric and prefibrillar aggregates. The fluorescence spectra of ThT (20 μM), **19c** (1 μM), and **18e** (1 μM) in the presence of either unincubated or incubated Aβ42 (5 μM) were recorded, showing for ThT and **19c** a small increase of the fluorescence intensity in the presence of an oligomeric form of Aβ42 and a larger enhancement with the fibrillar form of the protein. On the contrary, **18e** showed a very intense fluorescence signal in the presence of both a prefibrillar and a fibrillar form. Thus, all compounds act as sensors for fibrils, but **18e** can act as a sensor also for a prefibrillar form in an early stage of amyloid formation. In fact, **18e** has shown different emission spectra with prefibrils and a larger fibrillar protein, indicating a different binding mode toward the two different targets. This result confirms the potentiality of **18e** to discriminate the two conformational forms of Aβ42. In order to verify the ability of the OPEs to serve also as sensors for PA, which often correlated to AD pathology, the OPE spectroscopic responses were studied in the presence of α-synuclein fibrils. Oligomeric forms of α-synuclein were correlated to the disruption of mitochondrial activity, cellular membrane, and synapses. With this aim, the spectroscopic behaviors of OPEs toward an α-synuclein wild type and the three single mutant forms, A30P, A53T (involved in the early stage of PD), and E35K (which produces small oligomers), were analyzed. In particular, A30P aggregates more slowly than the wild type, while A53T seems to aggregate faster. ThT did not show significant changes in the excitation and emission, while on the contrary **19c** showed an increase of the intensities in the presence of all proteins except for A30P (probably due to a low abundance of oligomers) and was particularly intense for A53T. Compound **18e** in the presence of proteins showed similar behavior to **19c**, but the enhancement in this case was very pronounced (Figure 6b). All these results confirm the ability of the OPEs to act as sensors for prefibrillar and fibrillar aggregates that are already in the initial oligomeric form, thus representing a very powerful tool for the early diagnosis of AD and PD.

Very recently, Evans et al. [41] carried out a computational investigation about the binding dynamics of compounds **19c** and **18e** (Table 1) toward some Aβ oligomer models using classical all-atom molecular dynamics, in order to understand their interaction with amyloids. The studies were conducted with two β-sheet rich Aβ oligopeptides of 5- and 24-mer. The simulations showed that both positive and anionic OPEs can bind to the 5-mer protein, with a smaller number of molecules for **18e** than **19c**. In particular, **18e** formed mainly dimers, while **19c** was bound to protein through tetramers or pentamers, probably because of the higher electrostatic repulsions present in the more charged **18e**. Furthermore, the small 5-mer oligopeptide possesses a small binding surface, which limits the possibility of larger OPEs to bind to it. On the other hand, a higher number of OPEs, as a single molecule, as well as aggregates, were able to bind with 24-mer oligopeptides, both in the outer surface and at the end portions. In analyzing the aminoacidic presence at the protein surfaces, the kind and the charges of residue closer than 4 Å from OPE were determined. The binding sites for both OPEs presented mostly nonpolar residues (68% for **19c** and 77% for **18e**), while the ratio anionic/cationic residues was 0.25 for **19c** and 3 for **18e**, underling the importance of a correct electrostatic interaction in the binding. In addition, in analyzing the reasons of the fluorescence enhancement of OPEs after the binding event, the authors found a planarization of the aromatic backbone, with an important reduction in the dihedral angle distribution (close to 0°) when OPEs were bound to protein as complex (trimers or more). These observations partially explain the fluorescence intensity enhancement. Furthermore, the enhancement of the fluorescence is in part attributed to the desolvation of the water molecules in the ester-end group. For this reason, the number of molecules of water around the end groups of OPEs was calculated with and without the 24-mer protein. For the free OPEs, there are 15 water molecules, while for a single-binding protein/**19c**, a reduction was observed with a further decrease when OPEs were bound to protein through tetramers or pentamers, confirming a binding-inducing unquenching process. Thus, the present study confirms and supports the previous experimental results, showing that OPEs, due to the balance of electrostatic interaction, the hydrophobicity of the backbone, and the right size, can represent good platforms for sensing fibrils and prefibrils in an early stage of disease.

Another interesting application of OPE derivatives as sensors for saccharides was proposed in 2011 by Zhao et al. [42]. In this case, two kinds of OPEs that were conjugated with linear **20** and cruciform **21** π-frameworks (Table 1) and functionalized with phenylboronic acid were used in physiological conditions for the detection of saccharides with high sensitivity. In the synthesized OPEs, the triazole linker was able to act as a hydrogen-bond donor to stabilize a boron-saccharide complex, as confirmed by NMR studies. Meanwhile, the OPE aromatic core guaranteed a fluorescence response to the boron-saccharide complex aggregation state in solution. In particular, the best results were obtained for the cruciform-like OPEs **21**; in the absence of sugar, the OPEs are prone to aggregate, while the binding to saccharides results in a decrease of the aggregate’s dimensions, which in turn increases the fluorescence emission in a fluorescence turn-on sensing system.

## 4. Biocide Properties of OPEs

Drug (or multidrug) resistance is concerned with the ineffectiveness of the pharmaceutical treatments in blocking microbial infection. Through a series of mechanisms that are partly known, the pathogens recognize and modify the antimicrobial itself and thus neutralize it. The excessive use and misuse of antimicrobials in both common and hospital lives have accelerated this process. The development of resistance from pathogens suggests that the research of new efficient strategies against infections is still a challenge for researchers who care about public health. The research conducted on the antimicrobial properties of OPEs fits into this very complex but stimulating framework.

**Figure 7:**
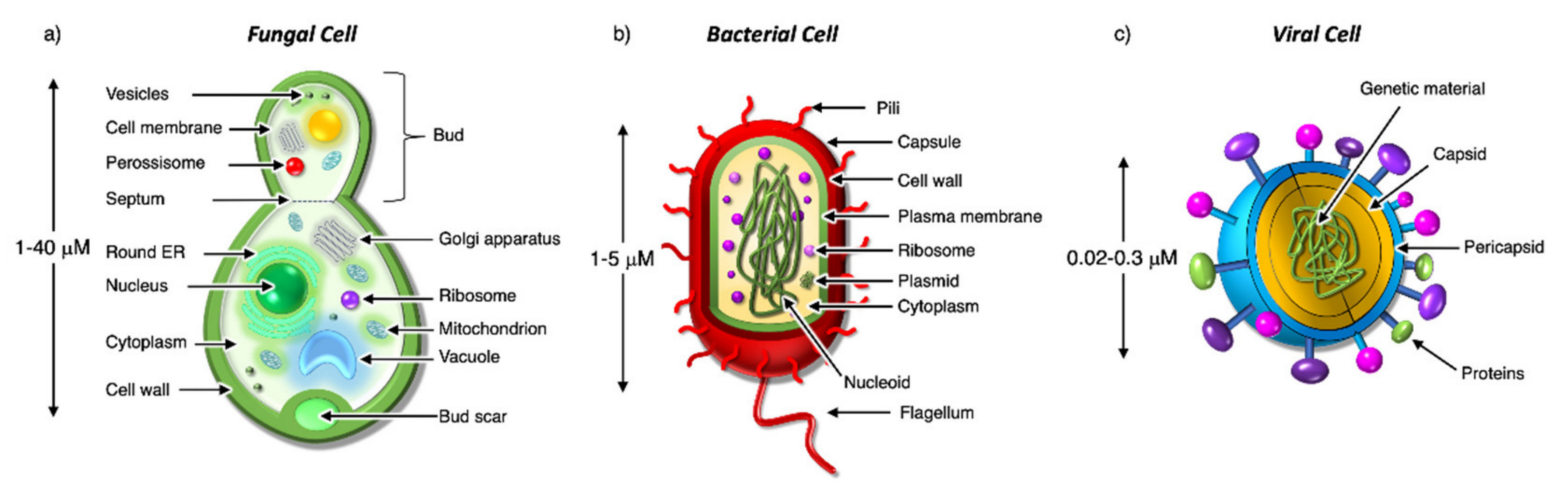
Schematic representation of some microorganisms: (**a**) fungal (**b**) bacterial, and (**c**) viral cells.

If readers consult the literature reported in this review, they may realize that much has been done by Whitten’s research group, but much more can be developed in terms of new OPE structures and improved antimicrobial properties. With the above in mind, this section comprises three main subjects: antibacterial, antifungals, and antivirals (Figure 7).

### 4.1. Antibacterial Properties of OPEs

From the discovery of penicillin (Fleming in 1945), bacteria have developed their resistance against antibiotics rapidly, causing the loss in control of hospital infections, essentially due to some particularly resistant strains. The acronym ESKAPE includes six nosocomial pathogens that exhibit multidrug resistance and virulence and render even the most effective drugs ineffective: *Enterococcus faecium*, *Staphylococcus aureus*, *Klebsiella pneumoniae*, *Aucinetobacter baumannii*, *Pseudomonas aeruginosa*, and *Escherichia coli* [43].

The antibacterial properties of OPEs have been mostly tested and studied on some of these pathogens and their resistant strains, and on some others, such as *Staphylococcus epidermidis*, an opportunistic bacterium usually found on skin and is paid little attention to. Because of the nonspecific antibacterial mechanism of action, it seems that it is difficult for bacteria to become resistant to OPEs, thus making them promising candidates for the development of novel drugs that are not subjected to bacterial resistance.

In Table 1, the structures of the OPEs that have been involved in the study of their antibacterial properties are reported. Almost all of them were synthesized by authors of research papers; only OPEs **22** is commercially available. Most of the studied OPEs are amphiphilic, possessing hydrophilic substituents at the side chain of the hydrophobic PE backbone. Although the different skeletons of OPEs, such as the length of their chain, can have a significant influence on the properties of these compounds, the mechanism of the attack of OPEs to bacteria has been described as very similar for all the examined oligomers.

**Figure 8:**
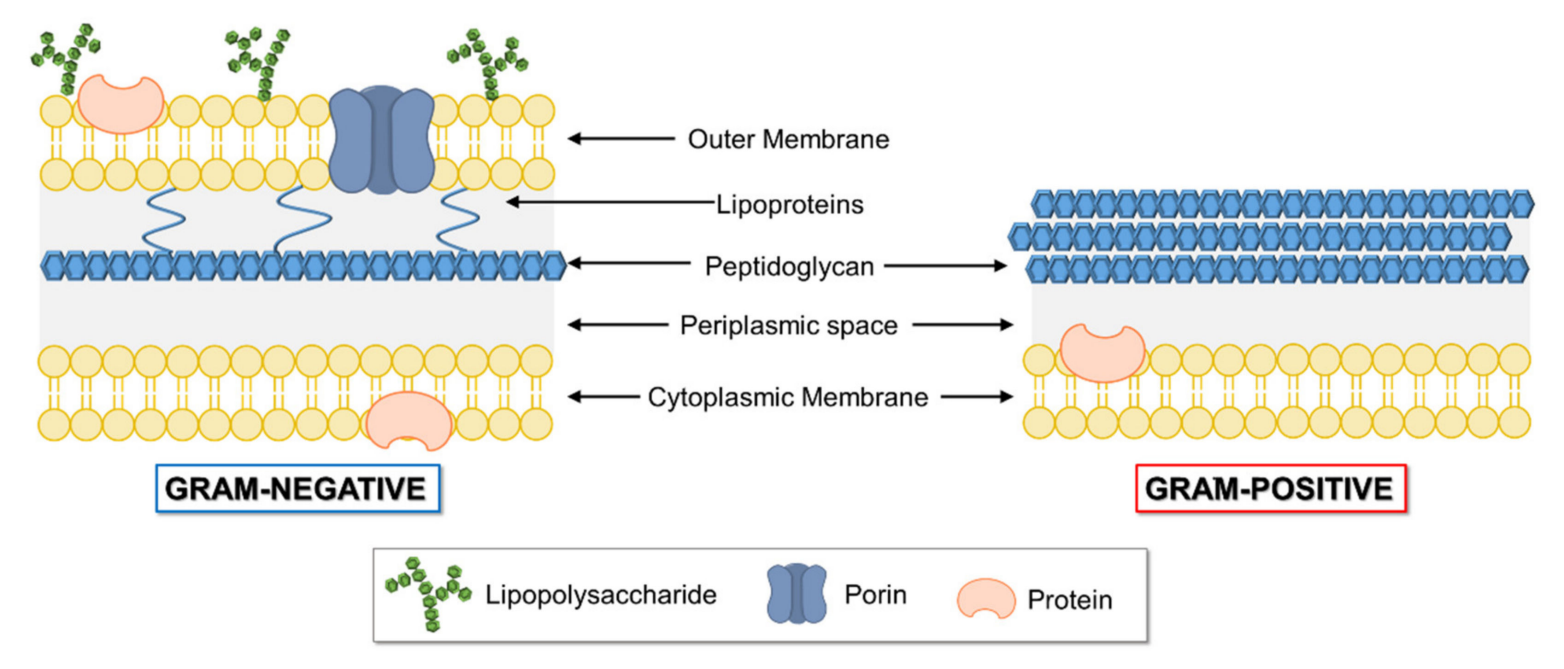
Gram-negative and Gram-positive external membranes.

Because of the structural complexity of the bacterial membrane (Figure 8) and its interactions with intra- and extracellular networks, the investigation on the mechanism by which OPEs exert their antibacterial properties, which concerns damages of bacterial membranes, is usually conducted on artificial model membranes [44]. Liposomes, with a lipid composition based on the differences between Gram-positive and Gram-negative bacteria and between mammalian and human erythrocyte membranes, have been widely employed to mimic how a bactericide interacts with these membranes. However, there is no shortage of studies in vitro where damages by OPEs to the bacterial membranes have been conducted on the bacterial surface and proven by scanning and transmission electronic microscopy (TEM and SEM) techniques. A recent in vivo investigation has been also carried out.

Moreover, the study of biocidal mechanism of OPEs has been usually conducted in parallel with two experimental conditions adopted: the dark [45] and the exposition to UV-light irradiations [46].

Generally, bacteria are classified as Gram-positive and Gram-negative depending on the results obtained with Gram’s test, a differentiation that is derived from their cell wall properties. Gram-positive bacteria, such as the most studied *Staphylococcus epidermis* (*S. epidermis*) or *Staphylococcus aureus* (*S. aureus*), have a cytoplasmatic membrane and a multi-layered and voluminous cell wall, essentially constituted by murein (peptidoglycans) that confers rigidity to the wall, whereas Gram-negative bacteria, such as *Escherichia coli* (*E. coli*) and *Pseudomonas aeruginosa* (*P. aeruginosa*), are characterized by the presence of an inner and an outer membrane and a thin murein wall between them (Figure 8). The outer leaflet of the external membrane is usually formed by phosphatidylethanolamine (PE), phosphatidylglycerol (PG), and cardiolipin (CL), such as that in *E. coli*, which has represented for many decades the standard model in the study of bacterial membrane composition for its simplicity [47].

**Figure 9:**
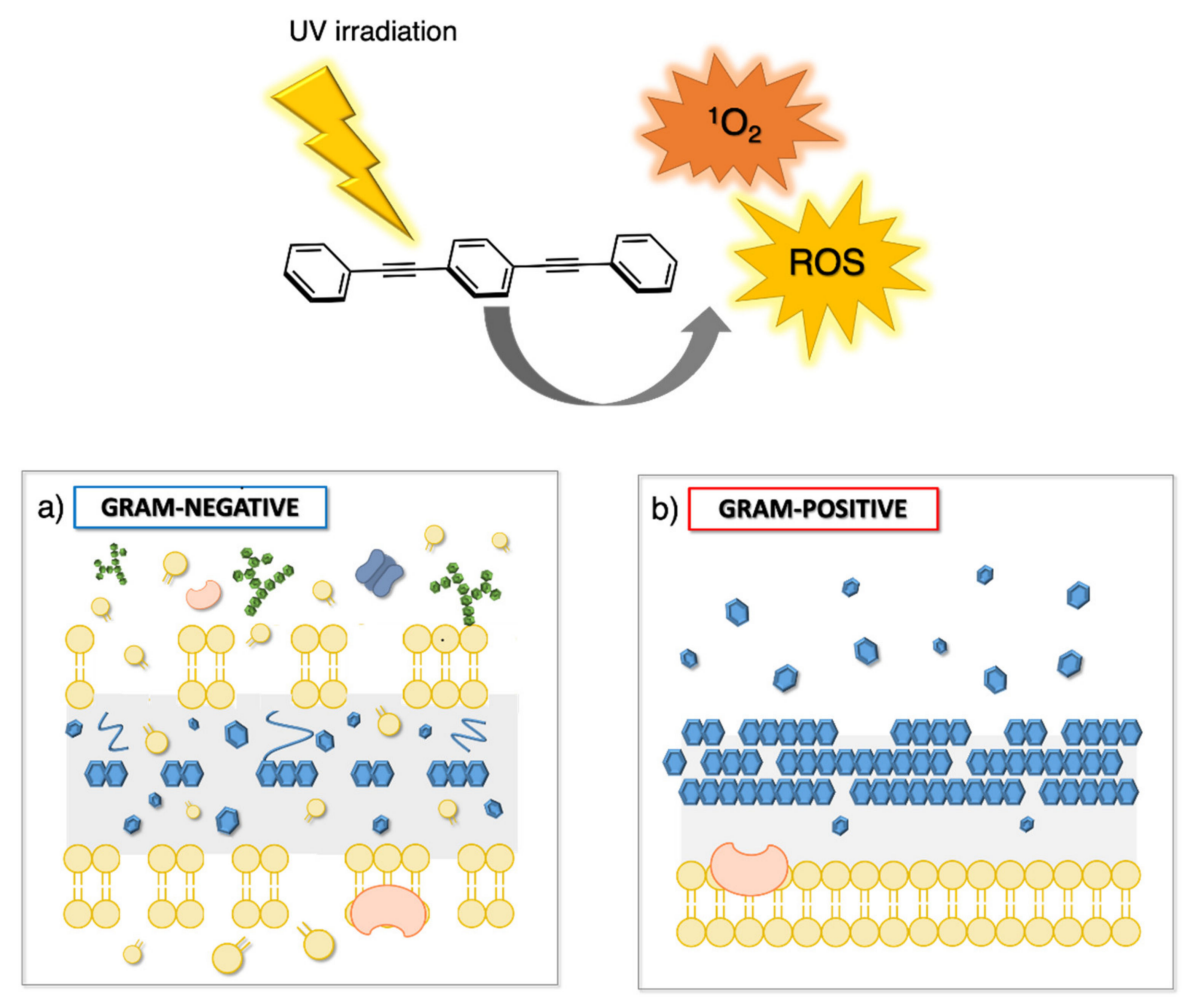
Schematic representation of the killing mechanism of Gram-negative and Gram-positive by OPEs.

OPEs have shown different antimicrobic effects in the dark against Gram-negative bacteria with respect to the Gram-positive ones. The lack of an outer membrane in Gram-positive bacteria and the presence of a thick cell membrane that is essentially composed of peptidoglycans allow for an easy access to molecules of molecular weights in the range of OPEs that exert higher antimicrobial activities against these microorganisms than Gram-negative bacteria. However, the cytoplasm of bacteria such as *S. epidermis* do not appear to be damaged by OPEs, which may be because they are not capable of penetrating the thick and negatively charged Gram-positive cell membrane. In this case, the disruption of the cell wall seems to be sufficient to ensure cell death. On the other hand, it has been well defined that in the dark, OPEs penetrate the bacterial cell wall of Gram-negative bacteria without causing disruption of such membrane and can reach the cytoplasm membrane quite easily (Figure 9). This perturbation correlates with their effective antimicrobial activity [10]. Membrane interaction is dependent on the molecular size (since compounds that are not large enough to cross the cell wall have negligible activity) and concentration of the oligomers [48]. Moreover, linear and positively-charged OPEs, such as OPE **23** in Table 1 with a rod-like structure, have shown to have a higher membrane penetration power than side-chain positively-charged OPEs (see for example OPEs **18d**-**f, 24d**-**f** in Table 1) but with a significant lowering of selectivity when a bacterial cell membrane was compared with a model of mammalian cell membrane that is composed of vesicles of PC lipids and cholesterol. Side-chain positively charged OPEs were found to be inactive against the model mammalian cell membrane [46]. Studies by classical molecular dynamics (MD) simulations [49,50] were conducted on linear end-only decorated OPEs (OPE **23b** in Table 1) and OPEs decorated at the core of the repetitive structure (OPE **18c** in Table 1), and for both of them it was proposed that antibacterial compounds strongly associate with the mimic lipid bilayer membrane (DOPE and DOPG phospholipids) in the first step. The insertion into the well-organized membrane induces a formation of water channels, which can be the reason for the disruption of the lipid wall. For OPE **23b**, the further formation of a pore was suggested, from which water leakage can occur.

Irradiation with the UV–visible light of bacteria in the presence of OPEs can efficiently produce the formation of singlet oxygen (^1^O_2_) and secondary reactive oxygen species (ROS) that induce, by oxidation, relevant damages or death of pathogens [44]. Lipids are not the major targets of their oxidation by OPEs in the presence of UV light, ^1^O_2_ and ROS can destroy other molecules on the surface or inside the bacteria. It has been demonstrated that some OPEs in the presence of UVA irradiation cause irreversible modifications to the proteins and plasmid DNA of the pathogens. Protein cross-linking and subsequent aggregation, critical changes in the structure, oxidation, strand breaking, and disruption of the structure of nucleic acids, are some of these modifications that can justify a great enhancement of the antibacterial properties of OPEs under UV irradiation [10]. The bacteria can be rapidly inactivated at very low doses of biocidal agents. Catastrophic damages to the morphology of Gram-positive and Gram-negative bacteria were observed after irradiation, whereas damages to the cytoplasm were registered just for the second ones, thus confirming that the thick and negatively charged bacterial wall of Gram-positive pathogens can prevent the penetration of OPE material into the cell interior [10].

Recently, Whitten’s research group studied the efficiency of the bactericidal effect of OPE **23c** against three bacteria that show antibiotic resistant strains [51]. The scope of their investigation was to establish the level and rate of dark- and light-activated antimicrobial activity. OPE **23c** was not as effective in the dark as under UV irradiation in killing the antibiotic resistant strains of *P. aeruginosa*, *S. aureus*, and *S. epidermis*. While some cationic OPEs were pointed out as very effective light-activated bactericide, it was shown that their prolonged exposure to UVA light can cause a drastic loss of their biocidal activity, leading to the formation of nonbiocidal photoproducts [52]. Molecular dynamic simulations revealed that the complexation of OPEs **23a,b** and **18a** (Table 1) with anionic surfactants such as sodium dodecyl sulfate (SDS) protects the oligomer backbone from contact with water and consequently from photolysis. Studies conducted on *E. coli* and *S. aureus* [53] by confocal microscopy revealed the stress response of both the bacteria cells to the OPE **23a**/SDS complex exposure after prolonged UV irradiation and confirmed the efficacy of such complexation. These data were useful for the development of photodegradation-resistant biocides and their application as sensors.

Zhou and Wang [14] reported the antimicrobial activity of OPEs, which essentially differ in their structures, for the presence of quaternary ammonium salts against tertiary amino groups (**24a,b** and **25a,b** in Table 1). Interestingly, the hydrophilicity and positive charge of OPEs **24a,b**—two structural characteristics that are usually considered needful—do not affect the antimicrobial activity of such molecules in a significant manner, since they appear less effective in killing *E. coli* and *S. aureus* bacteria than OPEs **25a,b** with tertiary ammonium, both in the dark and under fiber light irradiation. On the basis of this study, OPEs **25a,b** can be protonated in aqueous solution and attached to the bacterial wall by electrostatic interactions. Then, OPEs **25a,b** deprotonate and penetrate the bacterial membrane. The antimicrobial properties of these OPEs with tertiary amino groups are due to the synergic effect of bacterial membrane disruption and intracellular generation of singlet oxygen and/or ROS, determined by the combination of electrostatic and hydrophobic interactions present in compounds **25a,b**. The same authors report a study on a group of unsymmetrical OPEs that possess terminal tertiary and primary amino groups (OPEs **25c**, **26**, and **27a,b** in Table 1) [13]. Various experiments, such as lipid/water partition, destruction of mimic bacterial membranes, ROS generation test, and SEM imaging, were performed on Gram-positive and Gram-negative bacteria, and results confirmed the observations underlined in the paper before. The hydrophilicity/lipophilicity of these OPEs influenced the cell membrane penetration process, and the ROS, which was induced by irradiation, killed the bacteria.

Bacteria have many survival strategies, two of which are to exist in structural biofilms and to produce spores. The formation of bacteria biofilms is considered one of the major problems for hospital safety and public health. Their great resistance is due to the difficult penetration of an antibacterial, altered microenvironment within the biofilm, the different stress response of bacterial cells, and the formation of persistent cells. The action of a series of OPEs (OPEs **23a**,**c** and **28a**,**c** in Table 1) on *E. coli* biofilms was studied [54] using the minimum inhibitory concentration (MIC) and the minimum biofilm eradication concentration (MBEC) measurements, in the dark and under light activation. Once again, the results correlate the killing capacity of these antimicrobials to their structural features. An effective method to prevent the formation of bacteria biofilms was reported by López and Stiff-Roberts’s [55] research group, who described the deposition of commercially available oligomers **22** (Table 1) as thin films on solid surfaces and measured their antibacterial properties against *E. coli* bacteria, under UV exposure. These researchers used the innovative emulsion-based, resonant infrared, matrix-assisted pulsed laser evaporation (RIR-MARPLE) to form the OPE films, which guarantied enhanced bacterial attachment and biocidal efficiency compared to other film-deposition methodologies. The important advantage of the RIR-MARPLE technique is that a precise control of the surface morphology of the resultant OPE-film can be achieved without changing the chemical properties of the oligomer used. The same researchers perfected this methodology by incorporating to the OPE **22** films the thermally responsive poly(N-isopropylacrylamide) (PNIPAAm), which provided on demand the release of death bacteria from the surface of OPE films [56]. The antibacterial and bacteria-release properties of the OPE/PNIPAAm blended films were tested using two model bacteria: *E. coli* K12 and *S. epidermidis*. The blended films with an optimized composition showed the ability to kill the majority of *E. coli* K12 bacteria when under UVA exposure, and the death bacteria were easily removed from the films by the action of water.

**Figure 10:**
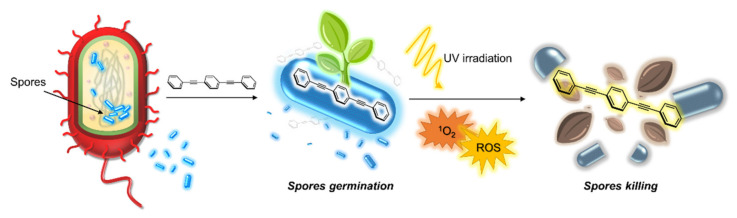
Schematic representation of spore death by the action of some OPE conjugates.

Bacteria produce spores because they can live in extreme environmental conditions. Spores are full-fledged cells that contain genetic material; they can have various forms and are found in a particular state of latent life. They are created when the cell is not in adequate environmental conditions. When spores finally find the right substrate and the right environment, the germination process begins. It lasts about 90 min and takes place in 3 phases: activation, initiation, and growth. The bacterial cell, in the form of a spore, is very long-lived; it can last between thousands and millions of years and suddenly stop the “hibernating” state and go back to being active when the right conditions arise. The spores resist very well to high and low temperatures as well as ultraviolet radiation due to their quiescence. They can be transported by atmospheric agents in different environments until they can find the right place to germinate. Many bacteria can produce spores, but some of them represent a problem for human health and are of considerable medical importance [57]. The dangerous spores of the bacterium *Bacillus anthracis*, which causes anthrax, for example, were used for a study on the effects of OPE **28a** (Table 1) on spore resistance [58]. In this study, *Bacillus atrophaeus*, a nonpathogenic surrogate of *B. anthracis*, was also tested. In both cases, OPE **28a** was found to kill the *Bacillus* spores under the irradiation of UVA light, but the surprising finding was to discover that the high killing power of the antimicrobial OPE stems from its ability to rapidly induce the germination of spores, rendering the bacteria vegetative cell vulnerable to the singlet oxygen and ROS species produced with irradiation (Figure 10). The germination of *Bacillus* spores as well as the death of germinated vegetative cells were confirmed in the dark experiments conducted by a flow cytometry assay. These results confirm that spores become more susceptible to biocidal agents upon their germination.

A very recent study [59] on the bactericidal properties of the unsymmetrical OPE **27a** (Table 1) underlined the capacities of such a molecule to act as significant biocidal agent even against multidrug-resistant (MDR) infections. This is the first in vivo study in which OPEs are involved. The results suggested that OPE **27a** has a good level of antimicrobial activity on both Gram-positive and Gram-negative MDR bacteria, confirming that the activity against the first ones was greater than that of Gram-negative bacteria. OPE **27a** could remove bacteria biofilms in a dose-dependent manner but did not inhibit biofilm formation under sub-MIC conditions, indicating that it cannot be used as preventive treatment for biofilm-associated infections. Furthermore, OPE **27a** was not toxic to mammalian cells at a concentration of ≤16 μg/mL, a concentration which could inhibit the growth of most resistant bacteria. Finally, the antibacterial effects of OPE **27a** were tested in vivo using a mouse model of skin burn infection caused by the inoculation of *P. aeruginosa* and *S. aureus*, the most opportunistic pathogens in this kind of infection. OPE **27a** could effectively prevent the inflammatory process, as confirmed by the increment of the necrotic epidermis of mice and the decrement of cytokines in their serum, in a dose-dependent manner.

### 4.2. Antifungal Properties of OPEs

Fungi are more complicated organisms than viruses and bacteria, as they are “eukaryotes” and are very similar to mammals in their cell structure. Both mammalian and fungal cells have membranes that are composed of chitin, glucans, mannans, and glycoproteins. In particular, the structural carbohydrate polymers glucan and chitin complement and reinforce each other in a dynamic process to maintain the integrity and physical strength of the fungal cell wall (Figure 7a). However, mammalian and fungal cells differ in their lipid composition [60]. Mammalian cells have a cholesterol-rich cell membrane, whereas fungal cells have a membrane that is primarily composed of ergosterol. Despite these differences, fungi are metabolically similar to mammalian cells and offer few pathogen-specific targets. Unicellular fungi (yeasts) are found in many different environments. Certain environmental fungi reproduce spores, such as bacteria, and these particles can enter human body through the lungs or on the skin. These fungi can be especially damaging for people with weakened immune systems, as they can spread quickly and damage many organs. To date, antifungal drug administration represents the first-line therapy against fungal infection. However, the numerous side effects induced by drugs toxicity and the appearance of drug-resistant strains emphasize the urgency of developing innovative therapeutic strategies [61]. The fungal cell wall is an attractive target for novel therapies, as host cells lack many cell wall-related proteins.

*Saccharomyces cerevisiae* (*S. cerevisiae*), the microorganism responsible for the most common type of fermentation, is one of the most intensively explored eukaryotic microorganisms, as *E. coli* is a model of prokaryotes. It was the first to be studied in the presence of some OPEs (OPEs **23b,c**, **24f**, and **28a** in Table 1) as antifungal agents; together with its ascospores and asci, these last as they are thick protective structures in which ascospores are enclosed [62]. In the dark, the studied OPEs exhibited moderate inactivation properties of the yeast vegetative cells, whereas under UV-light irradiation their antifungal properties were significant and dependent on the growth phase of the vegetative cells. Limited inactivation properties were observed for the ascospore and ascii. In the dark, most of the OPEs were inadequate to inactivate yeast ascospores, and under UV irradiation only OPE **24f** (Table 1) killed 95% of the ascospores—at least before their forced germination, which resulted in a weakening of their membrane wall and a consequent higher activity of OPEs.

**Figure 11:**
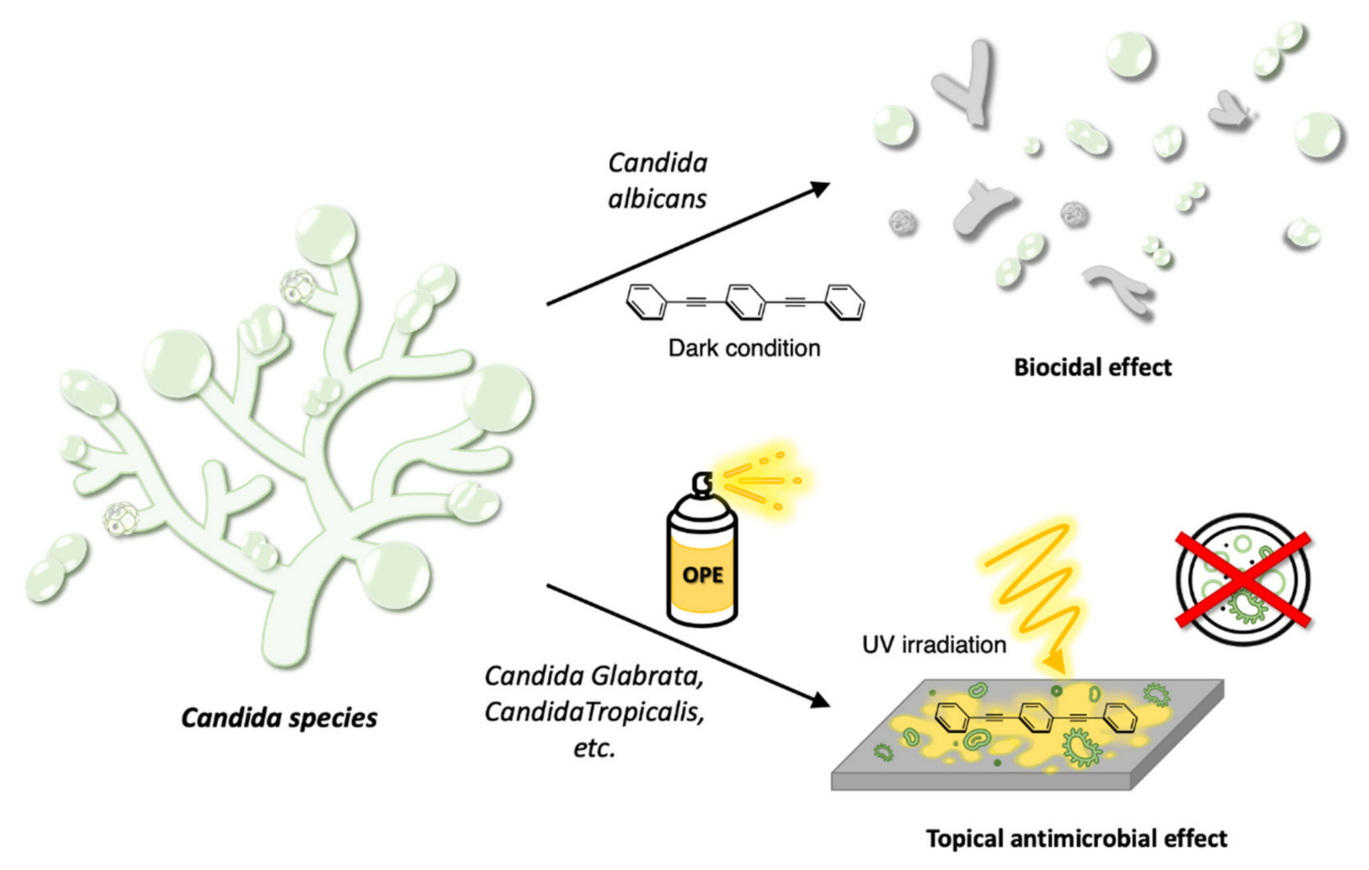
Cartoon representation of effect of some OPEs on *Candida* species.

Some clinical isolate strains of the *Candida* species, such as the polymorphic human pathogen *Candida albicans* (*C. albicans*), were subjected to OPE **23c** (Table 1) treatment with the intent of finding a new typology of disinfectants against the many bloodstream infections through contamination of special medical devices. OPE **23c** exerted strong biocidal effects against *C. albicans* even without light activation, but in general, all clinical isolate strains exhibited a significant level of killing after a period of light irradiation [63]. The partial resistance of *C. glabrata* can be due to the adaptation of the pathogen to growth in the host, thus causing changes in the cell wall and upregulation of the mechanisms that allow the pathogen to extend its life under adverse conditions. In any case, the authors underlined that OPEs could be used against *Candida* yeasts as topical antimicrobials in external medical devices as well as in wound dressing (Figure 11).

### 4.3. Antiviral Properties of OPEs

Viruses are obligate intracellular parasites: they are incapable of reproducing on their own and depend on the organisms they infect (hosts) for their very survival. When they find a cell to infect, the host cell is forced to rapidly produce thousands of identical copies of the original virus. If not inside the infected cell or in the process of infecting a cell, viruses exist in the form of independent particles (or virions) that are constituted of a protein coat called capsid, containing the nucleic acid DNA or RNA. The vast majority of viruses have RNA genomes. The capsid is made from proteins encoded by the viral genome, and its shape serves as the basis for morphological distinction [64]. Eukaryotic viruses can also have a membrane composed of lipids that surrounds the capsid and is called a pericapsid. An additional protein layer is sometimes present between the capsid and the pericapsid viruses (Figure 7c).

**Figure 12:**
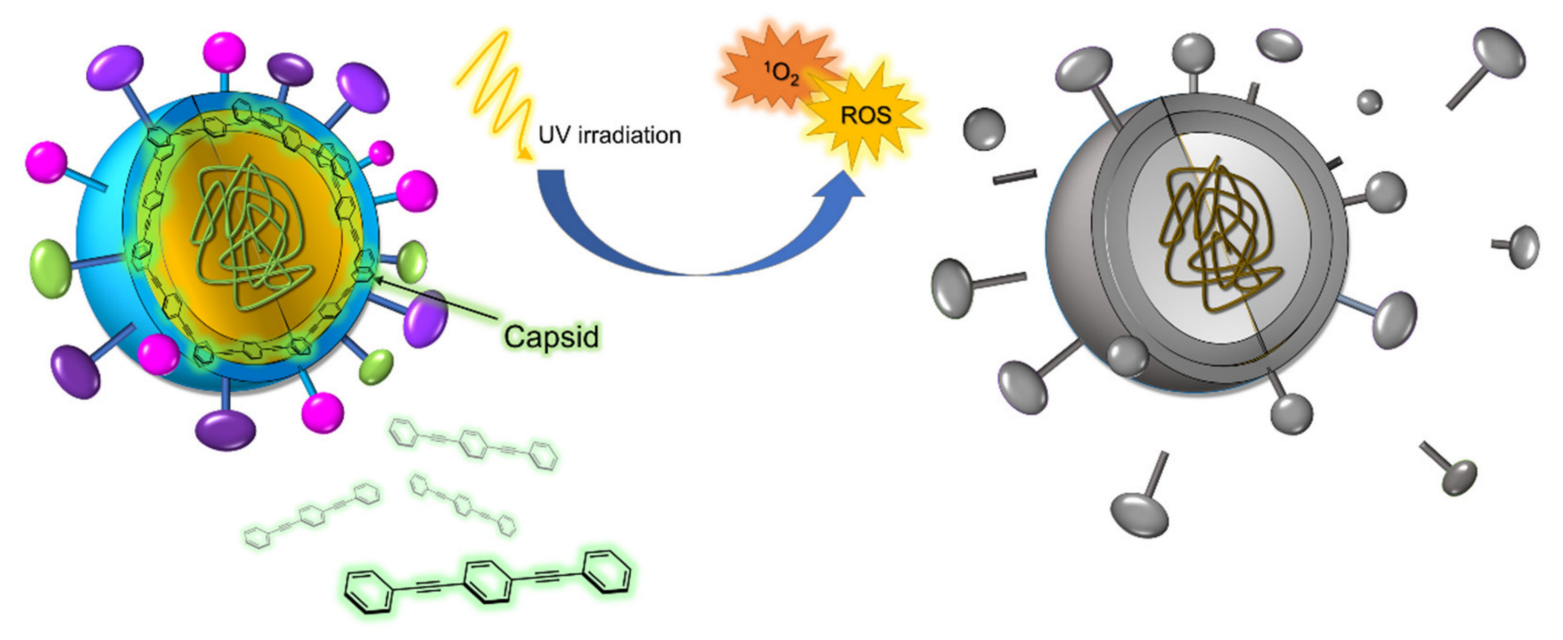
Illustrative image of viral attack by some OPE derivatives.

The first investigation on antiviral properties of some OPEs (OPEs **23b**, **24e,f**, and **28b** in Table 1) was conducted on two model viruses [65], i.e., the MS2 and T4 bacteriophages, which are commonly used for environmental pollution and virus detection studies. A bacteriophage, also known informally as a phage, is a virus that infects and replicates within bacteria. Bacteriophages are ubiquitous viruses that are found wherever bacteria exist, and they are among the most common and diverse entities in the biosphere. Most of the OPEs under study exhibited high inactivation activity against the MS2 phage and moderate inactivation activity against T4 bacteriophage when in the dark. This antiviral property can be in part due to the ability of OPEs to separate virus particles from the host cells and in part to the destruction of their external structure. When UV-light irradiation was used, the generation of corrosive reactive oxygen species induced strong damage to the capsid protein of the model viruses, thereby enhancing the antiviral activity of the studied OPEs (Figure 12). MD simulations were used to understand the interactions at the molecular level between oligomers **18b**, **19a**, **23c**, **24e**, and **29**, and the MS2 protein capsid, and the findings confirmed the experimental observations [66]. These simulations showed the strong binding of such OPEs to the MS2 coat, which was significantly disrupted. The binding was dominated by significant van der Waals interactions between the hydrophobic OPE skeleton and the capsid surface and the interactions between the OPE charged moieties and the charged residues on the capsid surface. It was also feasible that the strong nature of an OPE–capsid binding was influenced by the structural backbone of OPEs and the presence of charged groups on it. Moreover, the interaction of OPEs with the capsid surface may prevent the attachment by the virus and the infection of the host cells.

Very recently, Whitten’s research group [67] tested negatively and positively charged OPEs **19a,b** and **23d** against severe acute respiratory syndrome coronavirus 2 (SARS-CoV-2), the virus that caused coronavirus disease 2019 (COVID-19), the respiratory illness responsible for the COVID-19 pandemic. SARS-CoV-2 is a positive-sense single-stranded RNA virus that is highly contagious in humans. It is an enveloped virus containing several proteins that protect its internal RNA. The preliminary results of this study indicate that the tested OPEs **19a,b** and **23d** show biocidal activity against SARS-CoV-2 under near-UV light irradiation, and the antiviral activity of such compounds is due to a combination of the hydrophobic and electrostatic interactions between the oligomers and the SARS-CoV-2 proteins. It seems likely that OPEs **19a,b** and **23d** bind the viral proteins, and subsequently the generation of singlet oxygen by irradiation close to the RNA causes the inactivation of the virus. However, none of the tested OPEs exhibits antiviral properties in the dark, possibly because in this condition, the interactions between membrane proteins and oligomers are not so strong to cause the denaturation or bond-breaking of proteins.

## 5. Conclusions

The purpose of this review was to offer to the readers a portrait of the fluorescent wire-like OPEs engaged in a purely biological field. The initial thought that one may have when approaching the related literature is that everything has been experienced and everything obtained. This was the view we had when we started working on OPEs, and the question we asked ourselves was, what can we do that has not yet been done?

The history of the OPEs began as that of the younger brothers of the PPE, but as often as it happens in life, the minors appear to be cut out to take on the role that was previously designed for oldest. The synthesis of the repetitive phenyleneethynylene unit, opportunely decorated at the end- and side-chains and with different cores, is based on the Palladium(0)-catalyzed Sonogashira reaction for the coupling of small bricks that must be expertly prepared. However, as readers may observe in the Section 2 — “Synthesis of OPEs”, symmetrical and unsymmetrical 3-OPEs are the easiest to prepare by the convergent strategy of “one core with two stoppers” and exhibited the most significant properties in the biological field.

Like fluorescent probes such as fluorescein, rhodamine, or BODIPY derivatives, OPEs have been used for protein labelling in a classical fashion, introducing into their skeleton, for example, suitable groups able to bind proteins. Moreover, the amphiphilic character of some of these oligomers induces their self-assembly into nanoparticles or magnetic nanoparticles (using iron oxide nanocrystals decorated by amphiphilic OPEs), making them good tools for cellular bioimaging. The use of OPEs in the bio-optical field is not restricted to their use as fluorescent probes: they were easily transformed into fluorescent drug-carrying systems and sensors for amyloid fibrils, with performances comparable to those of ThT, a widely used dye for the detection of amyloids.

Certainly, the most intense studies conducted on OPEs have been concerned with their properties as antimicrobials and in particular, as antibacterials, despite the literature being mainly focused on in vitro studies (we could only find one work on in vivo applications). Gram-positive, Gram-negative, and multidrug-resistant bacteria, as well as bacterial spores and biofilms have been treated with several OPEs, and the results, as described in the dark and under UV irradiation, reproduced a portrait of very promising molecules for the development of new families of antibiotics. The antifungal and antiviral activity of OPEs have been also investigated, and the most recent results suggest that it is worth conducting more work in these two promising fields of applications. As matter of fact, the best results in both cases are obtained under UV irradiation, thus making OPEs assume the role of possible antivirals or antifungals for topical use, such as local disinfectants. On the other hand, the possibility of inserting specific moieties on the skeletons of OPEs in order to improve their affinity with microbials paved the way for significant upgrades in these two fields.

After our deep immersion in the OPE world, we now firmly believe that the possibility of the modulation of their skeleton in terms of substitution, length, and as a consequence their photophysical properties, makes the class of oligophenylene ethynylenes be extremely promising for their future exploitation in the biological field. Furthermore, their tendency to aggregate in supramolecular structures gives OPEs extra gear for the modulation of their photophysical features and, as a logical consequence, of their applicability in the mentioned field.

## Figures and Tables

**Table 1:** Reported OPEs and their bibliographic references.

Entry	Structure					Bibliographic References
**1**	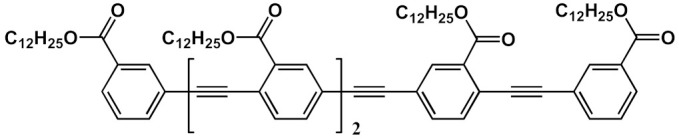					[9]
**2**	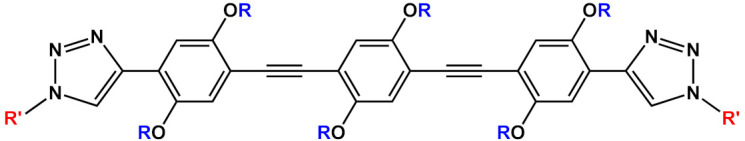	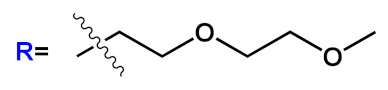	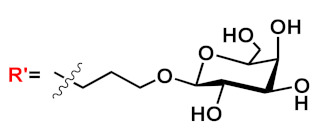			[5,6]
**3**	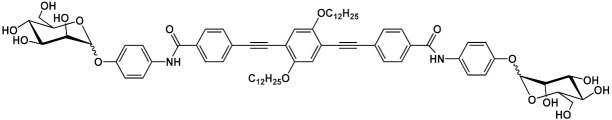					[12]
**4**	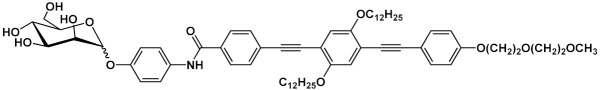					[12]
**5**	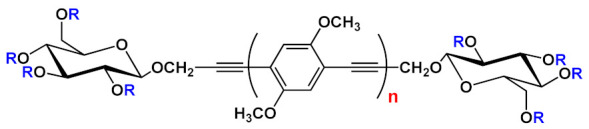	R=H	n=1	**5a**		[15,16,17]
			n=2	**5b**		
			n=3	**5c**		
		R=COCH_3_	n=1	**5d**		
			n=2	**5e**		
			n=3	**5f**		
**6**	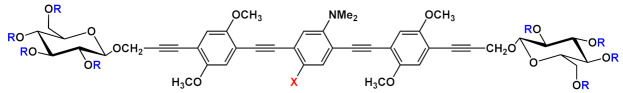	R=H	X=H	**6a**		[15,16,17]
			X=NO_2_	**6b**		
		R=COCH_3_	X=H	**6c**		
			X=NO_2_	**6d**		
**7**	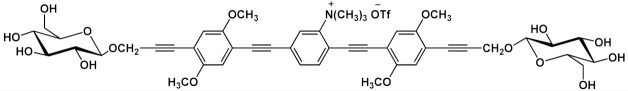					[17]
**8**	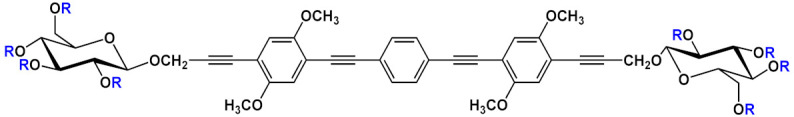	R=H	**8a**			[15,16,17]
		R=COCH_3_	**8b**			
**9**	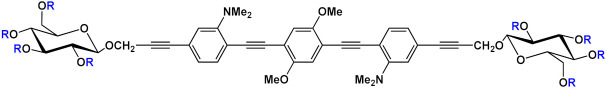	R=H	**9a**			[15,16,17]
		R=COCH_3_	**9b**			
**10**	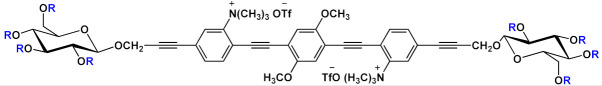	R=H	**10a**			[17]
		R=COCH_3_	**10b**			
**11**	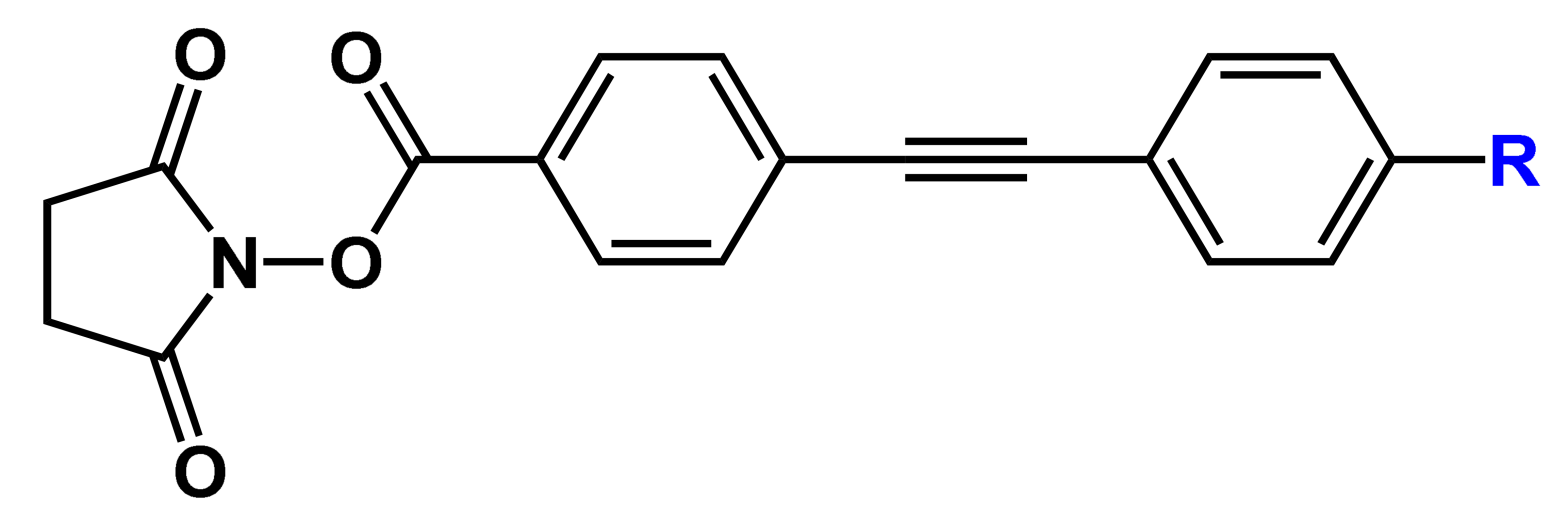	R=NH_2_	**11a**			[11]
		R=NMe2	**11b**			
		R=SMe	**11c**			
		R=OMe	**11d**			
		R=OH	**11e**			
		R=F	**11f**			
**12**	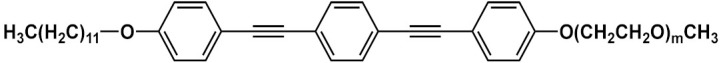					[24]
**13**	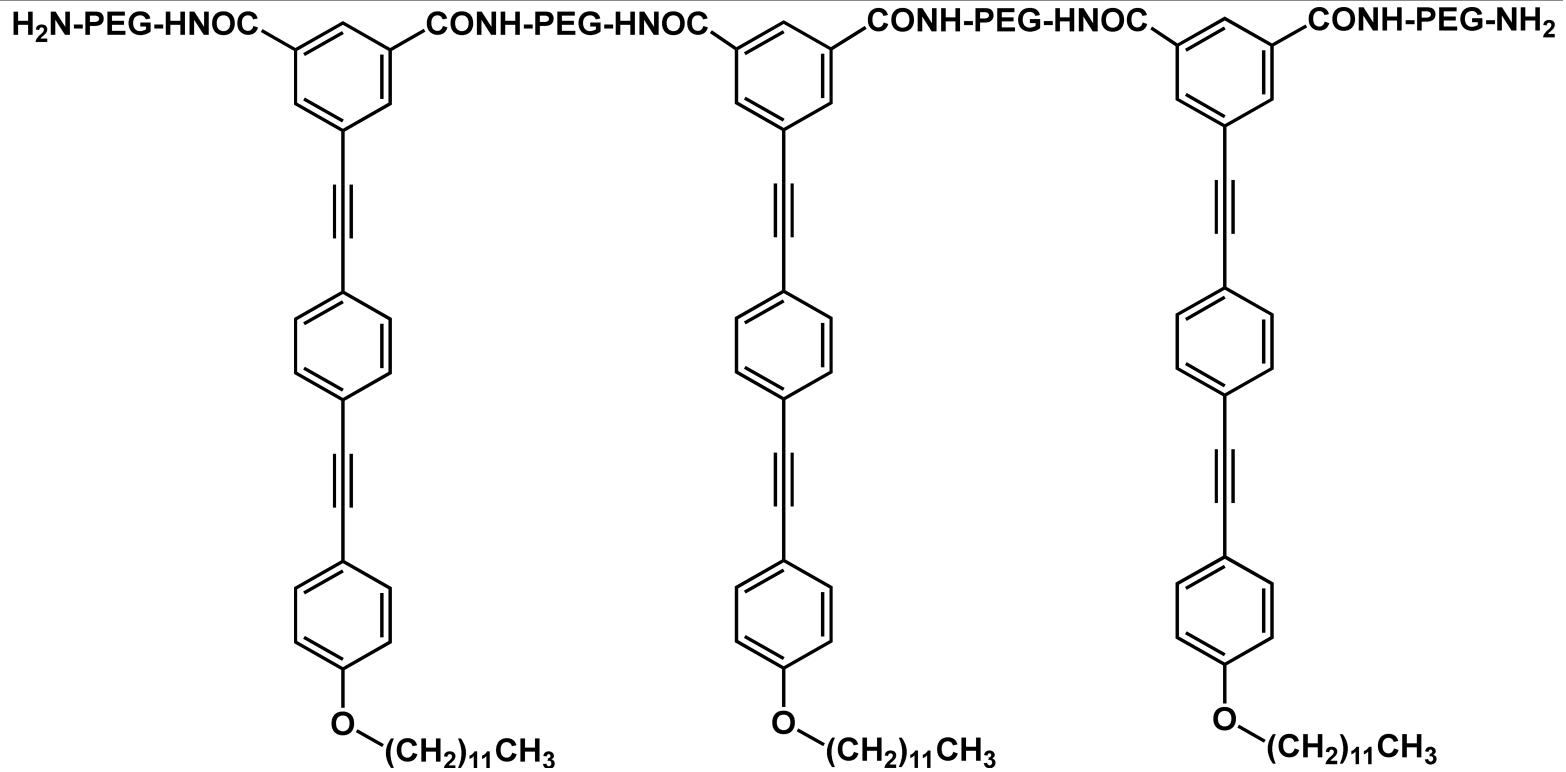					[25]
**14**	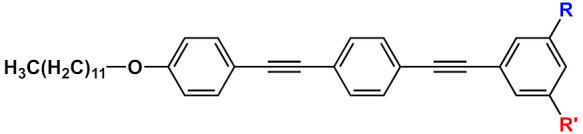	R=R’=CONH-PEG-NH_2_	**14a**			[26]
		R=CONH-PEG-NH_2_; R’=CONH-PEG-FA	**14b**			
**15**	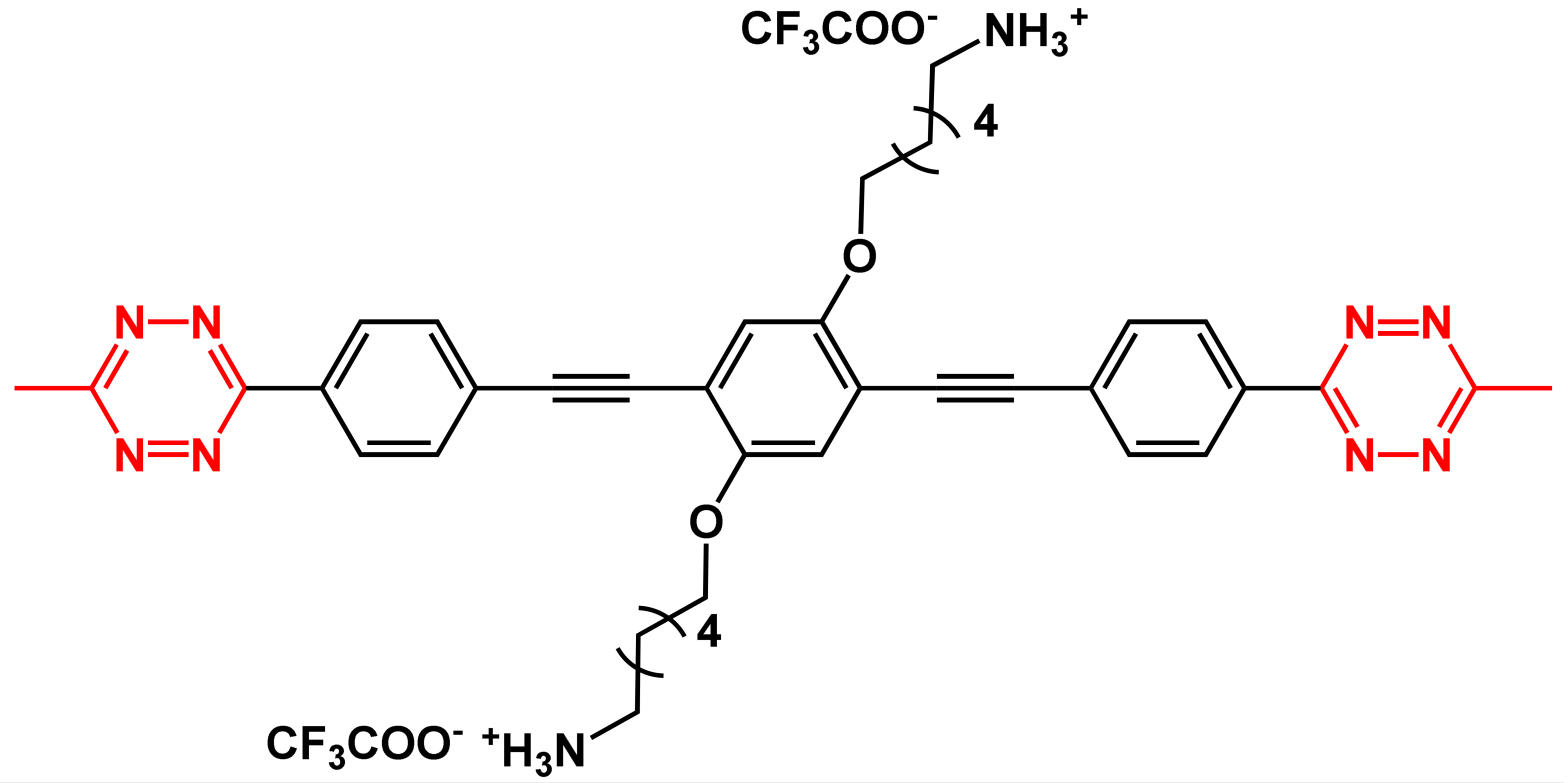					[27]
**16**	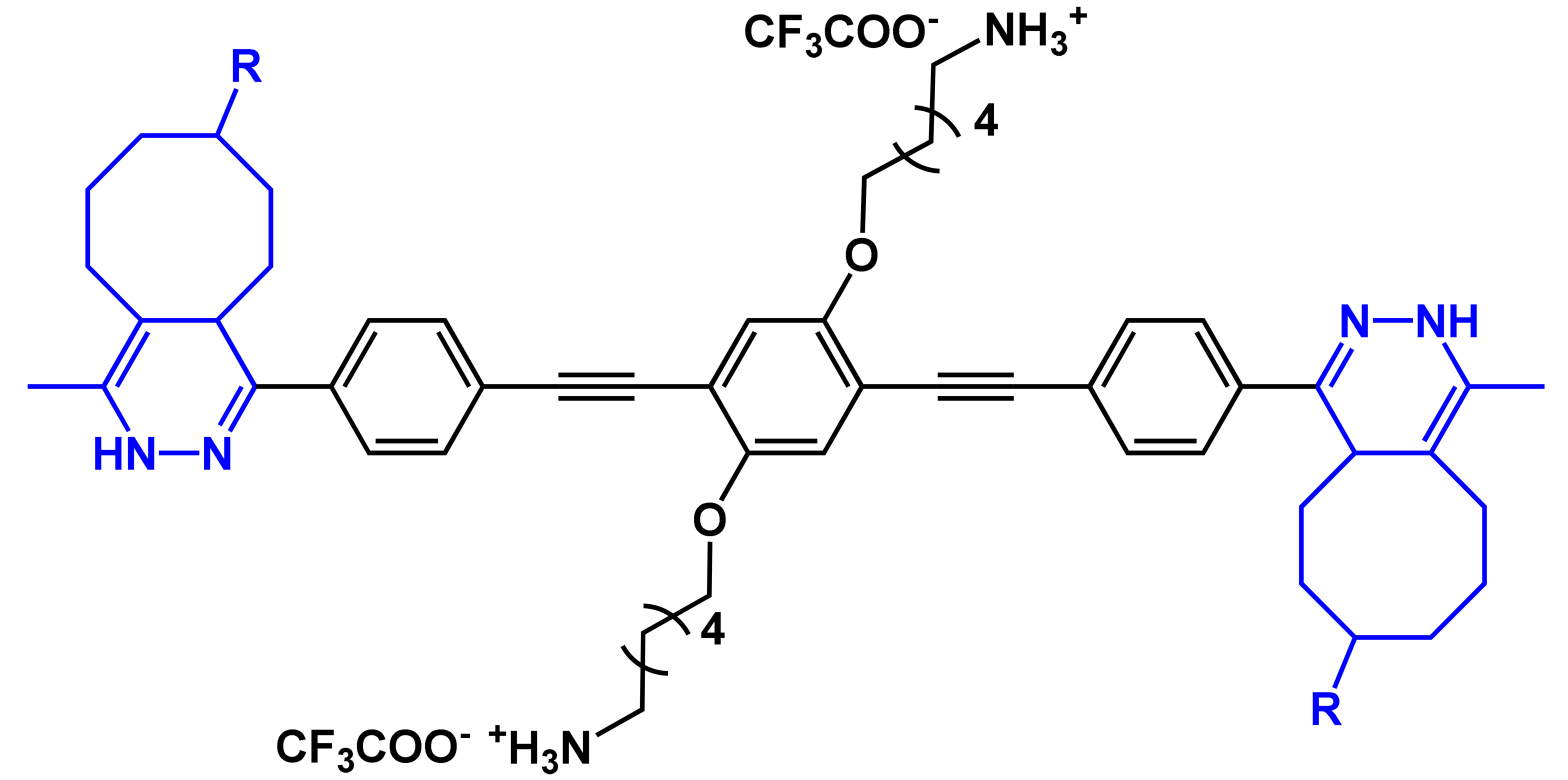	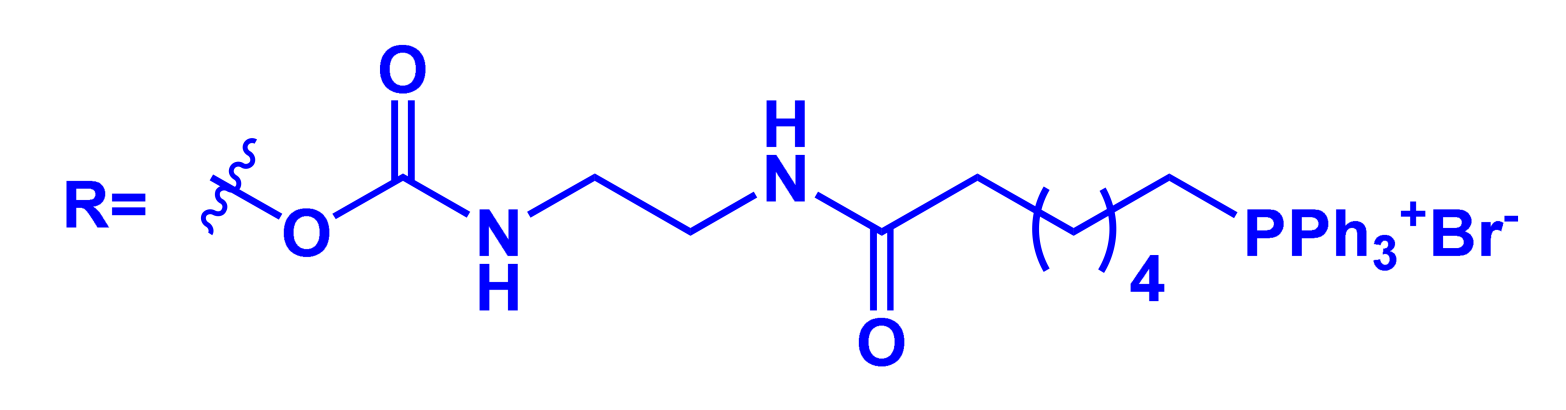				[27]
**17**	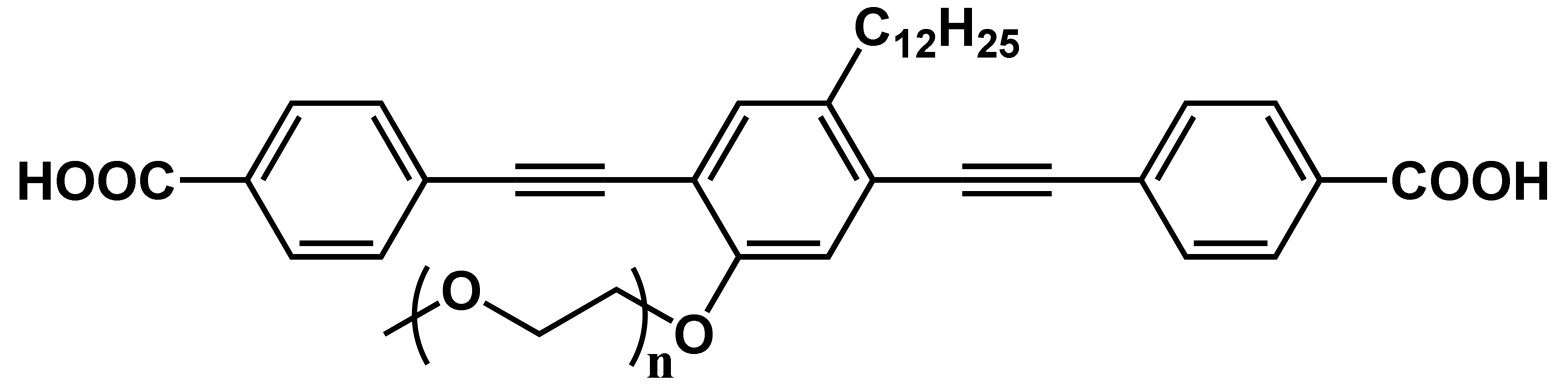					[28]
**18**	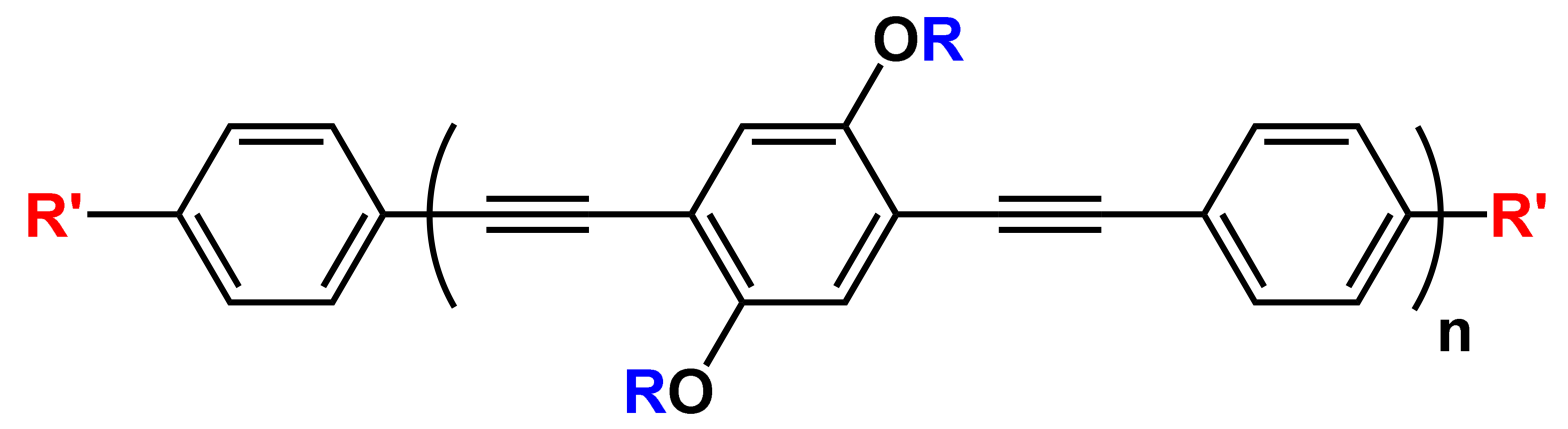	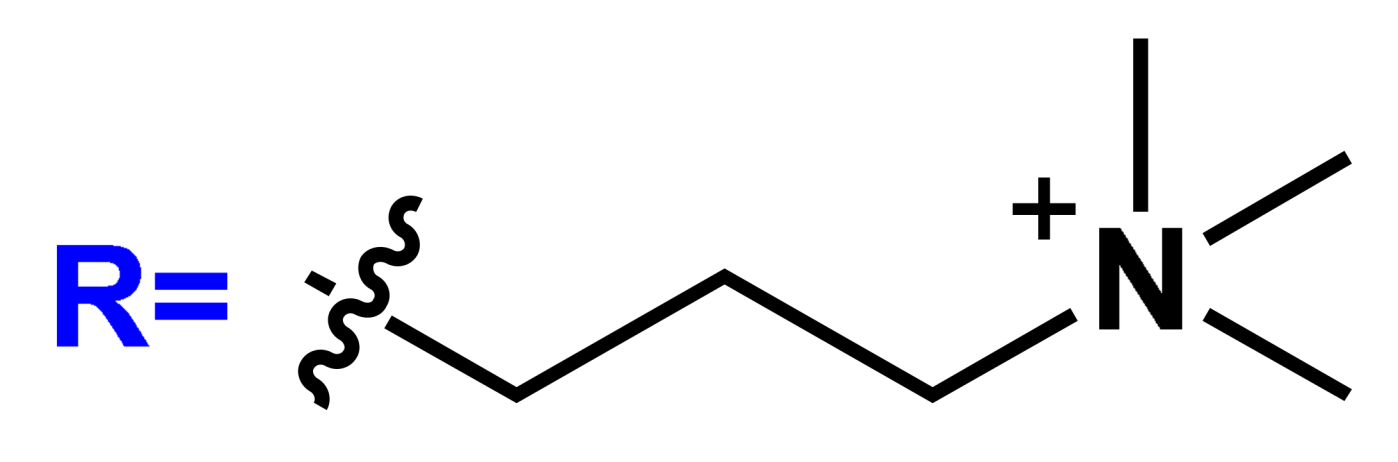	R’=H	*n*=1	**18a**	[34,37,39,40,41,46,49,50,51,52,53,66]
				*n*=2	**18b**	
				*n*=3	**18c**	
			R’=COOEt	*n*=1	**18d**	
				*n*=2	**18e**	
				*n*=3	**18f**	
**19**	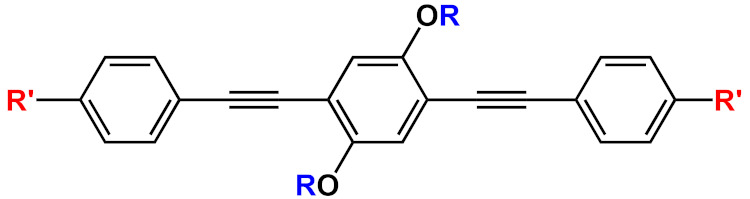	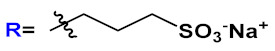	R’=H	**19a**		[34,37,39,40,41,66,67]
			R’=COOH	**19b**		
			R’=COOEt	**19c**		
**20**	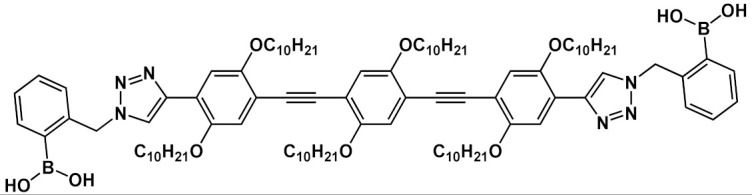					[42]
**21**	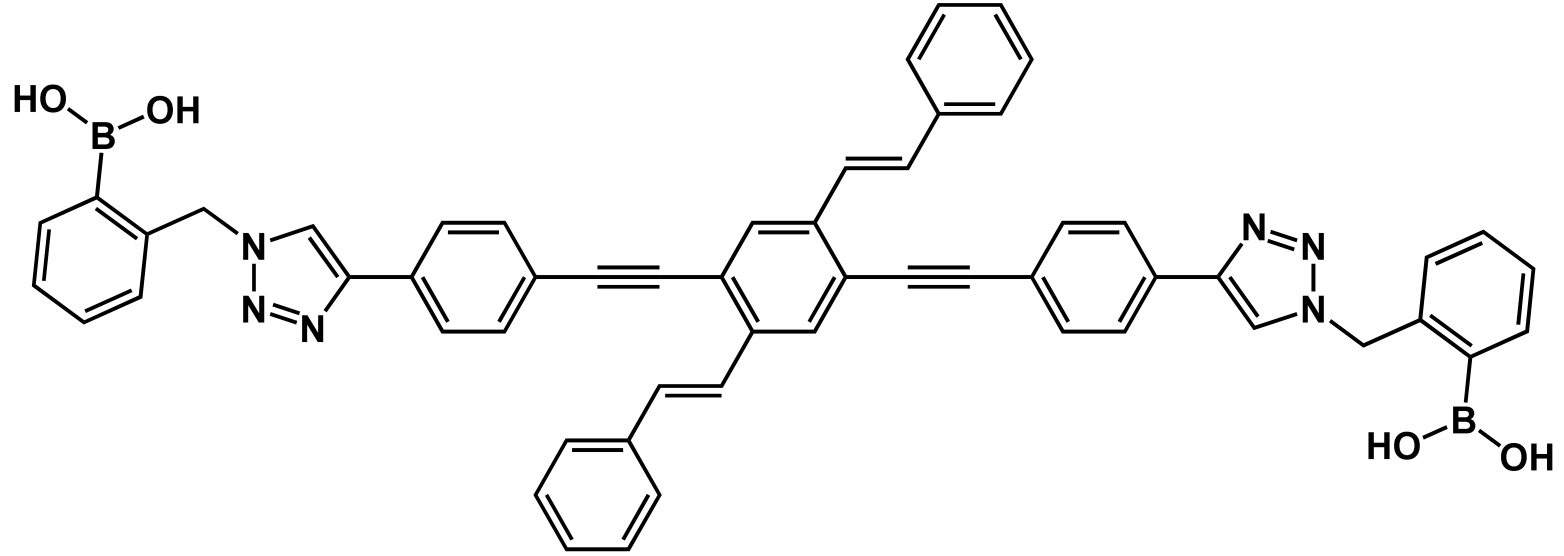					[42]
**22**	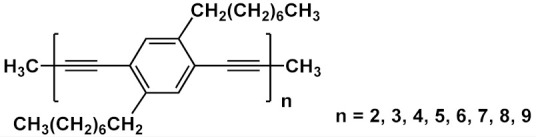					[55,56]
**23**	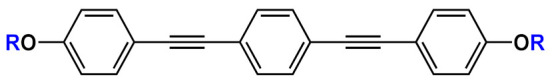	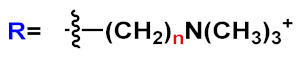	*n*=2	**23a**		[48,49,50,51,52,53,54,62,63,65,66,67]
			*n*=3	**23b**		
		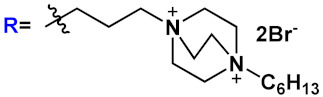	**23c**			
		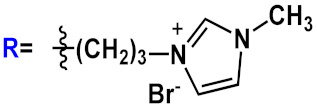	**23d**			
**24**	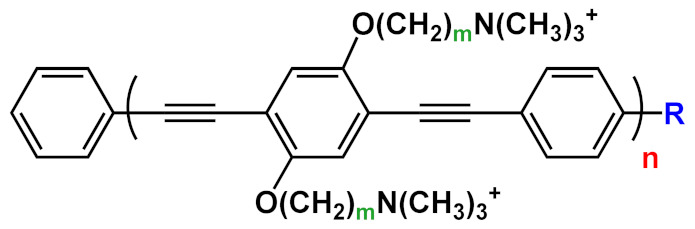	R=H	*n*=1	*m*=2	**24a**	[14,46,62,65,66]
			*n*=2	*m*=2	**24b**	
		R=COOEt	*n*=1	*m*=3	**24c**	
			*n*=2	*m*=3	**24d**	
			*n*=3	*m*=3	**24f**	
**25**	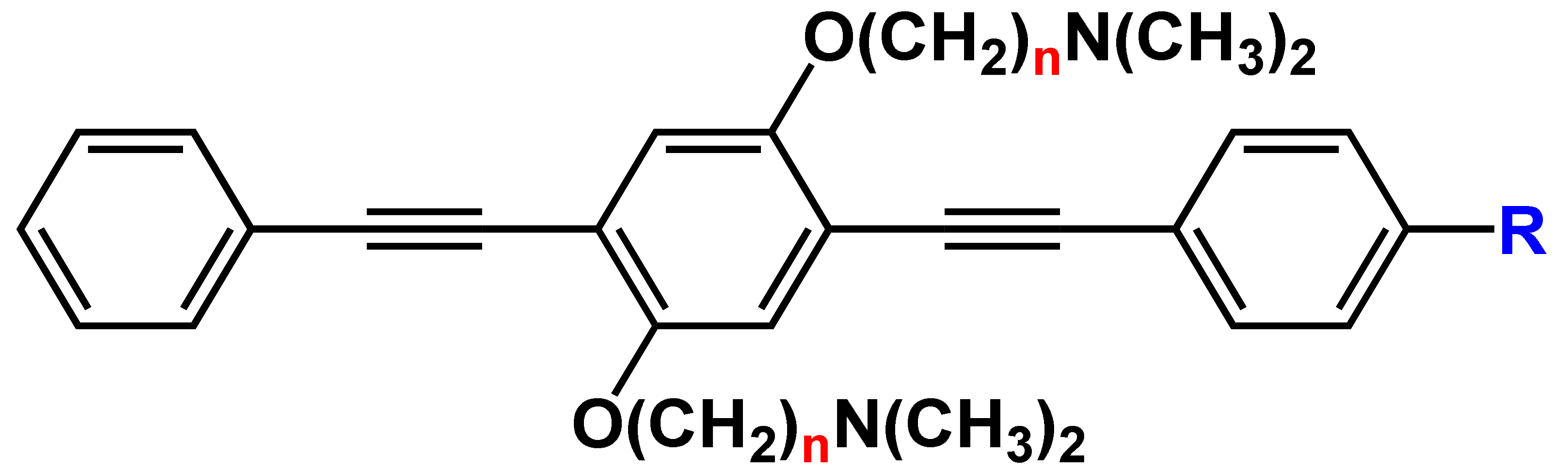	R=H	*n*=2	**25a**		[13,14]
			*n*=3	**25b**		
		R= O(CH_2_)_2_NH_2_	*n*=3	**25c**		
**26**	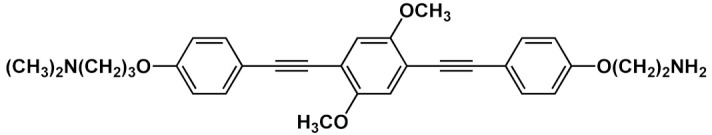					[13]
**27**	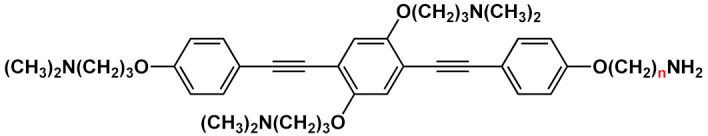		*n*=2	**27a**		[13,59]
			*n*=3	**27b**		
**28**	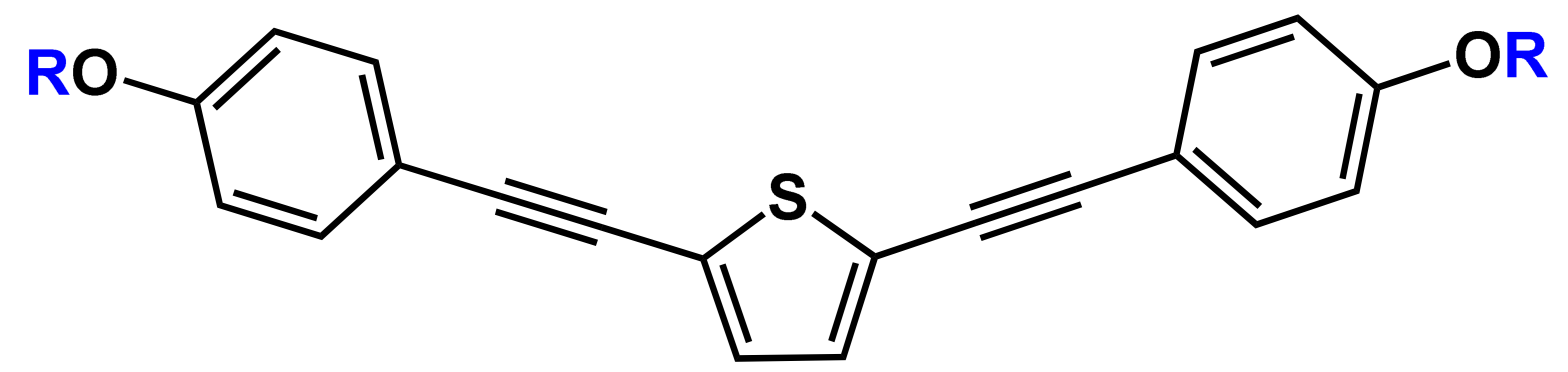	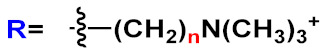	*n*=2	**28a**		[54,57,62,65]
			*n*=3	**28b**		
		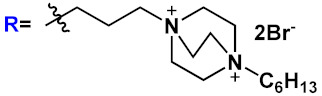	**28c**			
**29**	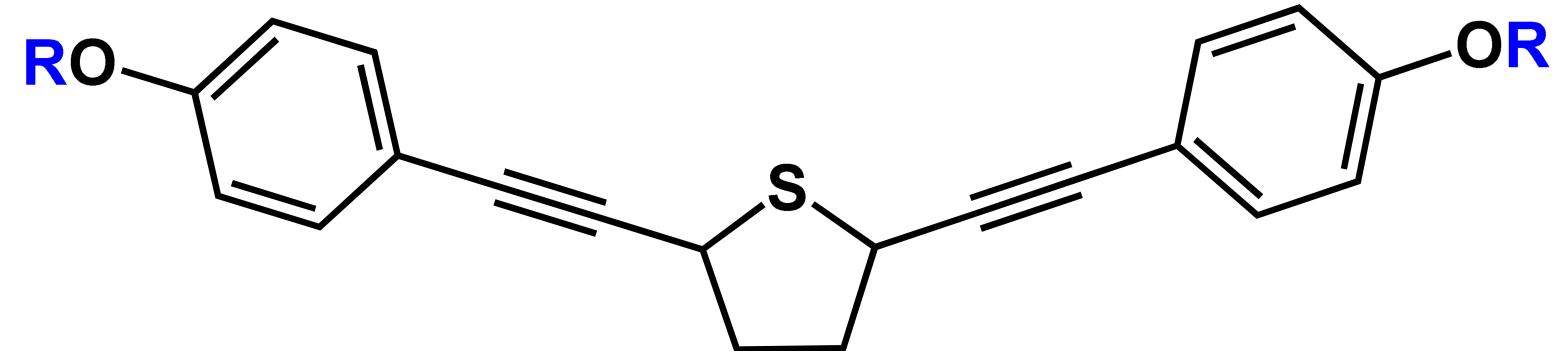	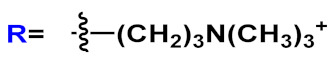				[66]
